# Boosting selective nitrogen reduction to ammonia on electron-deficient copper nanoparticles

**DOI:** 10.1038/s41467-019-12312-4

**Published:** 2019-09-26

**Authors:** Yun-Xiao Lin, Shi-Nan Zhang, Zhong-Hua Xue, Jun-Jun Zhang, Hui Su, Tian-Jian Zhao, Guang-Yao Zhai, Xin-Hao Li, Markus Antonietti, Jie-Sheng Chen

**Affiliations:** 1 School of Chemistry and Chemical Engineering, Shanghai Jiao Tong University, Shanghai, 200240 P. R. China; 2 Department of Colloid Chemistry, Max Planck Institute of Colloids and Interfaces, Wissenschaftspark Golm, Potsdam, 14424 Germany

**Keywords:** Electrocatalysis, Materials for energy and catalysis

## Abstract

Production of ammonia is currently realized by the Haber–Bosch process, while electrochemical N_2_ fixation under ambient conditions is recognized as a promising green substitution in the near future. A lack of efficient electrocatalysts remains the primary hurdle for the initiation of potential electrocatalytic synthesis of ammonia. For cheaper metals, such as copper, limited progress has been made to date. In this work, we boost the N_2_ reduction reaction catalytic activity of Cu nanoparticles, which originally exhibited negligible N_2_ reduction reaction activity, via a local electron depletion effect. The electron-deficient Cu nanoparticles are brought in a Schottky rectifying contact with a polyimide support which retards the hydrogen evolution reaction process in basic electrolytes and facilitates the electrochemical N_2_ reduction reaction process under ambient aqueous conditions. This strategy of inducing electron deficiency provides new insight into the rational design of inexpensive N_2_ reduction reaction catalysts with high selectivity and activity.

## Abstract (web summary)

Electrocatalytic nitrogen reduction is promising for ammonia production, but electrocatalysts are limited by low efficiency and high cost. Here, the authors report electron-deficient copper nanoparticles, induced by rectifying contact with polyimide, for selective reduction of nitrogen to ammonia.

## Introduction

The exploration of electrochemical N_2_ fixation into ammonia, sustainable fertilizers for crops or energy carriers in the hydrogen economy^1,2^, has drawn tremendous interest due to the gentle and ambient process and low energy requirements^3–5^. In nature, only certain nitrogenase bacteria are able to selectively break the nonpolar triple bond, which is the most important process in the natural nitrogen cycle^6^. Pioneering works applying various noble metal-based N_2_ reduction reaction (NRR) catalysts (e.g., Au, Pt, Ru, Ag)^7–10^ as well as several recent examples of transition metals^11–15^ and carbonaceous catalysts^16–18^ have already demonstrated the extraordinary advantages of these heterogeneous electrocatalysts, although the overall efficiency remains unsatisfactory. Further exploring novel strategies to elevate the selectivity (Faradaic efficiency) to NH_3_ and production rates of NRR catalysts, which are inexpensive and abundant, still remains challenging but highly alluring for the possible decentral NH_3_ generation on any scale under mild conditions.

Here, we report an effective strategy to boost the NRR activity of less active copper nanocatalysts via modulating the electron density of Cu nanoparticles over a significant potential scale to simultaneously depress the hydrogen evolution reaction (HER) activity and elevate the NRR activity for a higher Faradaic efficiency and yield rate of NH_3_ (17.2 µg h^−1^ cm^−2^) in addition to a turnover frequency (TOF) value of 0.26 h^−1^. Textural analysis sufficiently demonstrates that the Mott–Schottky effect^19–23^ leads to an electron redistribution at the interface of polyimide (PI)^24^ and copper and thus controls the work functions of Cu species for more feasible N_2_ reduction, giving rise to a new high TOF value of 0.26 h^−1^, which is higher than that of noble metal-based NRR catalysts.

## Results

### Fabrication and structure of Cu/PI catalysts

**Fig. 1:**
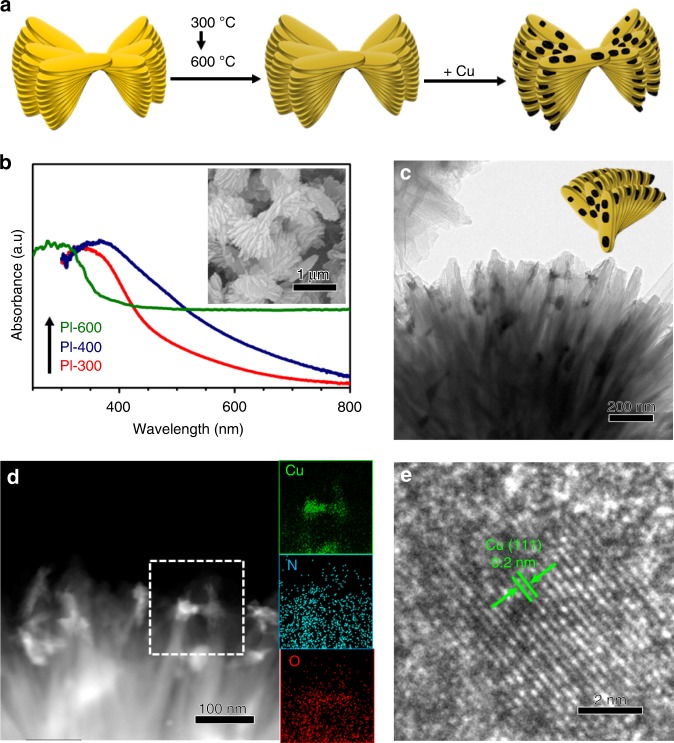
Fabrication and structure of catalysts. **a** Synthetic procedure of typical Cu/PI catalyst. **b** Ultraviolet-visible (UV-vis) spectra of bare PI samples. Transmission electron microscopy (TEM) **c**, high-angle annular dark-field (HAADF) **d**, and high-resolution TEM (HRTEM) **e** images of Cu/PI-300. **Insets: b** Scanning electron microscopy (SEM) image of PI-300; **d** Energy dispersive X-yay (EDX) mapping images of the selected area

The synthetic process for the Cu/PI catalysts is depicted in Fig. 1a (for experimental details, please see the Methods). A modified solvothermal method was applied to prepare PI nanoflowers, which were further condensed at 300 °C (PI-300), 400 °C (PI-400) and 600 °C (PI-600) to tailor the conjugating degrees and used as supports for depositing Cu nanoparticles via a wet impregnation method^23^. The color change in the PI supports from deep yellow to black directly reflects the gradually narrowed band structures, which have been further confirmed by their UV-vis spectra (Fig. 1b). The chemical structure of the PI support was verified by solid-state nuclear magnetic resonance (SSNMR) spectroscopy (Supplementary Fig. 1). After the introduction of Cu components, the flower-like morphology (Fig. 1b inset) of the PI supports with the thin nanosheets as the primary subunits remains unchanged, as revealed by the scanning electron microscopy (SEM) (Supplementary Fig. 2) and transmission electron microscopy (TEM) observation (Fig. 1c and Supplementary Fig. 3–5), as did the chemical structure as confirmed by the Fourier transform infrared (FT-IR) analysis (Supplementary Fig. 6). TEM (Fig. 1c) and high-angle annular dark-field (HAADF) (Fig. 1d) images further demonstrate the integration of Cu nanoparticles inside the PI flower. The similar mean sizes of Cu nanoparticles around 30 nm (Supplementary Fig. 7) for all Cu/PI samples exclude possible size effect on their catalytic activity. Further Energy dispersive X-ray (EDX) mapping (Fig. 1d insert) images exhibit the homogenous distribution of N and O atoms along the PI supports and also verify the formation of Cu-containing nanoparticles. A typical crystalline lattice distance of 0.2 nm (Fig. 1e), attributed to the (111) facet of metallic Cu, further confirmed by its X-ray diffraction (XRD) patterns (Supplementary Fig. 8), prove the coexistence of Cu and PI in the Cu/PI samples. X-ray photoelectron spectrum (XPS) results (Supplementary Fig. 9) not only provide a chemical composition of Cu/PI containing only C, N, O and Cu but also exclude the presence of lattice oxygen in possible copper oxides or hydroxides (Supplementary Fig. 10).

### N_2_ reduction reaction performance of Cu/PI catalysts

**Fig. 2:**
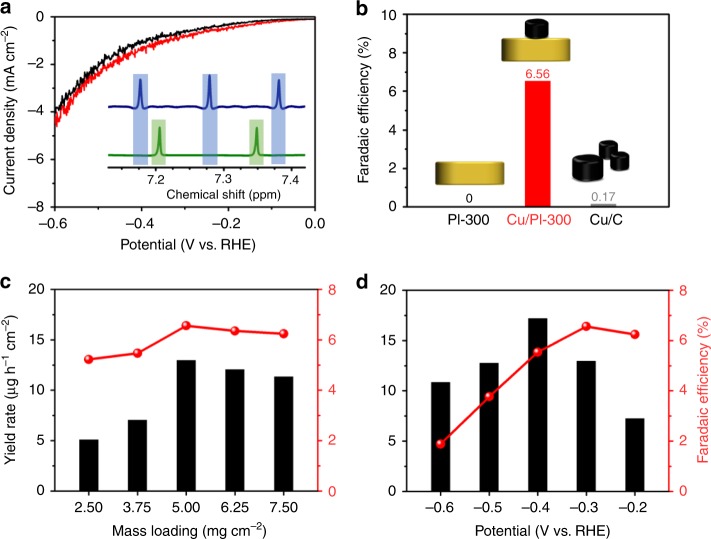
N_2_ reduction reaction performance of the electrocatalysts. **a** The linear sweep voltammogram (LSV) curves of Cu/PI-300 (catalyst loading: 5 mg cm^−2^) measured at a scan rate of 10 mV s^−1^ under the Ar and N_2_ atmosphere. **b** The Faradaic efficiencies of bare PI-300, Cu/PI-300, and Cu/C for NH_3_ generation at −0.3 V vs. RHE within 6 h. The Faradaic efficiencies (spheres) and NH_3_ yield rates (bars) of Cu/PI-300 with varied catalyst loadings at −0.3 V vs. RHE **c** or with a fixed catalyst loading (5 mg cm^−2^) at different work potentials **d** within 6 h. **Insets: a** ^1^H NMR spectra of both ^14^NH_4_^+^ and ^15^NH_4_^+^ produced from the NRR reaction using ^14^N_2_ and ^15^N_2_ gas respectively

We initially examined the possibility of the Cu/PI hybrids for use as NRR electrocatalysts in basic solution (0.1 M KOH) at room temperature. At first glance, the Cu/PI electrode (exemplified with Cu/PI-300 materials) offered a larger current density in N_2_ flow than the reference measurement in Ar (Fig. 2a), revealing a possible selectivity towards N_2_ reduction. Indeed, a standard electrocatalytic reaction over the Cu/PI-300 electrode with an optimized Cu content of 5% (Supplementary Fig. 11 and Table 1–2) resulted in the best NRR Faradaic efficiency of 6.56% at a potential of −0.3 V vs. RHE (Fig. 2b and Supplementary Fig. 12). Carbon cloth (current collector), bare PI-300 electrode (Fig. 2b) and CuO_x_/PI-based electrode (Supplementary Table 2) were inert under the given conditions. It should be noted that the yields of ammonia were determined by both colorimetric method (Supplementary Fig. 13) and ion chromatography method (Supplementary Fig. 14). ^15^N isotope labeling experiments (Fig. 2a insert and Supplementary Fig. 15) confirm the electrocatalytic reduction process of the N_2_ gas over the Cu/PI-300 electrode into corresponding ammonia.

The mass loadings of Cu/PI-300 on the carbon cloth also slightly change the Faradaic efficiencies and the NH_3_ yield rates, and this condition was optimized as 5 mg cm^−2^ to afford the highest Faradaic efficiency (6.56%) and NH_3_ yield rate (12.4 µg h^−1^ cm^−2^) at a potential of −0.3 V vs. RHE (Fig. 2c and Supplementary Fig. 16). Cu/C catalyst (Supplementary Fig. 12), as a control sample of Cu nanoparticles prepared from the same method with Cu/PI, provide a Faradaic efficiency of only 0.17% (Fig. 2b) and a rather low yield rate of NH_3_ (~ 0.7 µg h^−1^ cm^−2^) (Supplementary Table 2), attesting to the essential contribution of the Cu/PI combination to the high selectivity to NRR. The NH_3_ yield rate of the Cu/PI-300 electrode with a catalyst loading of 5 mg cm^−2^ could be further elevated to 17.2 µg h^−1^ cm^−2^ at an optimized potential of −0.4 V vs. RHE (Fig. 2d and Supplementary Fig. 17).

### Effect of electron deficient Cu

**Fig. 3:**
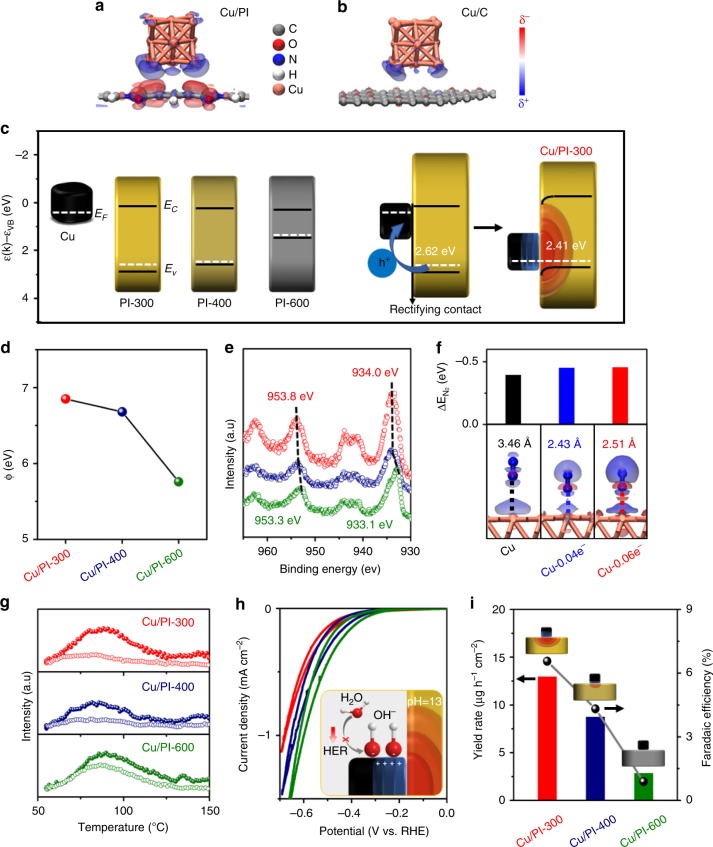
Effect of electron deficient Cu nanoparticles on the catalytic performance. EDD stereograms of **a** Cu/PI and **b** Cu/C models. **c** Experimentally estimated work functions and band structures of Cu and PI components (left) and schematic diagram of rectifying contact of Cu and PI hybrid (right), resulting in electron rich (red) and deficient (blue) areas at their interface. The estimated work functions from UPS analysis **d** and X-ray photoelectron spectroscopy (XPS) Cu 2p spectra **e** of Cu/PI samples. **f** The calculated adsorption energies of N_2_ molecules on pristine Cu (Cu) and electron-deficient Cu (Cu-0.04e^−^and Cu-0.06e^−^) surface. **g** The N_2_-temperature programmed desorption (TPD) results of Cu/PI catalysts (spheres) and corresponding bare PI supports (circles). **h** The cyclic voltammograms (CVs) of Cu/PI electrodes (catalyst loading: 1 mg cm^−2^) at a scan rate of 5 mV s^−1^ in Ar-saturated 0.1 M KOH. **i** The NH_3_ yield rates and Faradaic efficiencies of Cu/PI electrodes at −0.3 V vs. RHE. **Insets**: **f** EDD stereograms of N_2_ molecules on pristine Cu (Cu) and electron-deficient Cu (Cu-0.04e^−^and Cu-0.06e^−^) surface. **h** the deactivation mechanism of the HER process over electron deficient Cu nanoparticles in Cu/PI-300; **i** proposed electron localization at the Cu-PI boundaries

To understand the supporting effect on the NRR activity of supported Cu nanoparticles, we calculated the electronic structures of Cu clusters on PI and carbon supports via density functional theory (DFT) simulation. The electron density difference (EDD) (Fig. 3a, b) and Hirshfeld charge (Supplementary Fig. 18) results illustrate that the PI as a semiconductor support could attract more electrons from the Cu cluster (0.06 for each Cu atom) than that by carbon support (0.02 for each Cu atom) in Cu/C model, indicating a rectifying contact between Cu and PI semiconductors. Indeed, the programable band structures of PI-300, PI-400 and PI-600 samples (Fig. 3c) were further estimated on the basis of ultraviolet photoelectron spectroscopy (UPS) results (Supplementary Fig. 19), UV-vis absorption spectra (Fig. 1b and Supplementary Fig. 20) and Mott–Schottky plots (Supplementary Fig. 21). The thermal condensation process largely elevated the valence band positions of PI supports for PI-300 and PI-400 with higher synthetic temperatures, while their conduction bands were slightly elevated simultaneously. As presented by the SSNMR and FT-IR results (Supplementary Fig. 1 and 6), the PI-600 sample was highly condensed into organic carbons with a narrower band gap and also an improved conductivity. Accordingly, the estimated work functions of the PI supports decrease from PI-300 via PI-400 to PI-600. It is thus reasonable that PI-300 could attract more electrons from the deposited Cu nanoparticles due to the rectifying Mott-Schottky effect^25,26^ at the Cu/PI interface, which was reflected by the greatly enhanced work function of Cu in Cu/PI-300 (Fig. 3d) estimated from the UPS analysis results (Supplementary Fig. 22). Furthermore, the gradual shifts of typical Cu 2p_3/2_ XPS peaks (Fig. 3e) to higher energy was attributed to the gradually increased numbers of electrons donated by Cu particles of the PI supports with even higher work functions (Fig. 3d). It doubly verified that PI-300 could attract more electrons from the deposited Cu nanoparticles according to the larger shift of Cu 2p_3/2_ XPS peak for Cu/PI-300 (Fig. 3e). It should be noted that the typical Cu 2p_3/2_ peaks of copper oxides was estimated to be centered at 935.0 eV (data not shown), directly excluding the presence of CuO as the main component in all Cu/PI samples. The gradually decreased electron density in metallic Cu in Cu/PI samples from Cu/PI-600 to Cu/PI-300 (Fig. 3d, e) again confirm that Cu is the electron donor, as depicted in the right side of Fig. 3c, making the Cu nanoparticles more “noble”.

### Density functional theory calculations

We next carefully analyzed the actual role of the electron deficiency of Cu nanoparticles in activating N_2_ molecules and boosting their NRR activity. The polarization of adsorbed N_2_ molecules was gradually enhanced by Cu surface with lowered electronic density which was well presented by the obvious differences in the electron density difference (Fig. 3f insert) and Hirshfeld charge (Supplementary Fig. 23) of each N atom after changing the catalytic surface from the pristine Cu to the electron-deficient Cu (Cu-0.04e^−^ and Cu-0.06e^-^) models. The strengthened interaction with N_2_ on Cu surface with lowered electronic density was also well reflected by the enhanced adsorption energy (Fig. 3f). Such a strong interaction between N_2_ and Cu/PI dyads was further confirmed by the N_2_ adsorption-desorption and N_2_-TPD analysis results (Fig. 3g and Supplementary Fig. 24–25). More pronounced electron-deficiency of Cu nanoparticles from Cu/PI-600 via Cu/PI-400 to Cu/PI-300 leads to gradually increased N_2_ adsorption capacities for 0.6, 2.6 and 4.1 times as compared to the values of corresponding bare PI supports (Supplementary Fig. 25c). The lowest surface area of Cu/PI-300 among all Cu/PI samples (Supplementary Fig. 24) directly demonstrate the main contribution of electron-deficient Cu to its high N_2_ adsorption capacity.

**Fig. 4:**
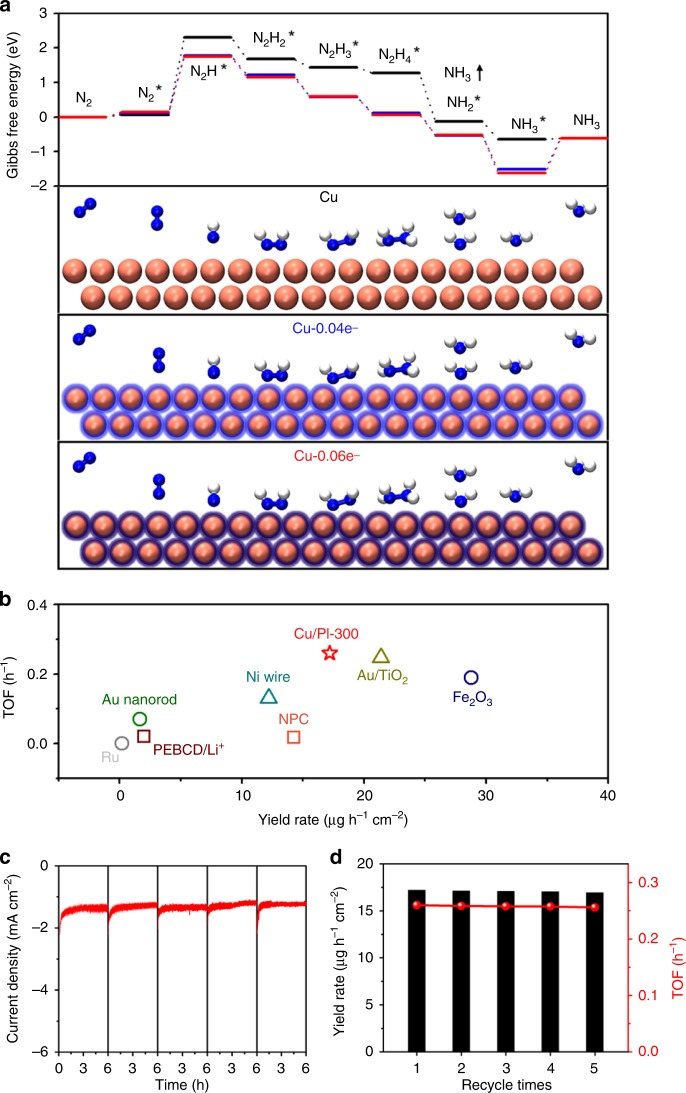
Density functional theory calculations and stability. **a** Calculated absorption configurations (bottom) and corresponding Gibbs free energy diagrams of each step of NRR process on pristine Cu (black), Cu-0.04e^−^ (blue) and Cu-0.06e^−^(red) models. **b** The turnover frequency (TOF) values and NH_3_ generation yield rates of Cu/PI-300 and benchmarked NRR electrocatalyst in the literature (details listed in Table S3). The i-t curves **c** of five runs of 6-h NRR reaction over a reused Cu/PI-300 at −0.4 V vs. RHE and corresponding NH_3_ yield rates and TOF values **d**

Moreover, depressing the HER process during NRR is another aspect to ensure the final selectivity of a catalyst. For the HER process in basic electrolyte conditions, the adsorption of water molecules and desorption of OH^−^ usually dominate the entire mass transfer process. An electron-deficient surface of Cu nanoparticles obviously generates a strong electrostatic interaction with the OH^−^ anions (Fig. 3h inset), which is undesirable for HER on the Cu-centers in base solution. As a result, Cu/PI-300 exhibited the worst HER performance (Fig. 3h) among all Cu/PI samples in this work. The much higher Tafel slope for HER over the Cu/PI-300 catalyst compared with other controls (Supplementary Fig. 26) directly confirm the negative effect of the electron-deficiency induced in the Cu nanoparticles on HER, which is, on the other hand, highly preferred for the improvement of NRR performance for gradually elevated ammonia yields (Fig. 3i). More importantly, the addition of first hydrogen atom to pre^−^adsorbed N_2_ (*N_2_) as the rate^−^limiting step and the following steps, according to the calculated Gibbs free energy change (ΔG) of the NRR (Fig. 4a and S27) are also gradually facilitated by the Cu clusters with even lower electronic density as reflected by the remarkably lowered ΔG (from 2.30 eV on pristine Cu via 1.76 eV on Cu-0.04e^-^ to 1.60 eV on Cu-0.06e^-^). The dissociation step from *NNH_4_ to *NH_2_ proceed automatically again due to the largely reduced free energy by the electron-defecient Cu surface.

Both experimental and theoretical results demonstrate the key effect of electron density of metallic Cu nanoparticles on the promoted NRR process. As a results, the Cu/PI-300 nanocomposite containing electron-deficient Cu nanoparticles is the first example of Cu-based nanocatalysts for electrochemical NRR with a Faradaic efficiency of 6.56%, while CuS (Supplementary Fig. 28), as the best-in-class Cu-based NRR catalyst in the literature, yields a Faradaic efficiency of only 0.18%^27^. Furthermore, the NH_3_ generation yield rate of Cu/PI-300 (17.2 µg h^−1^ cm^−2^) is also among the highest levels reported for state-of-the-art NRR electrocatalysts, far surpassing the reported Cu-based NRR electrocatalyst and comparable to noble metal-based counterparts (Fig. 4b and Supplementary Table 3). It should be noted that utilization of more noble Cu nanoparticles as the catalytically active centers in Cu/PI-300 provides a TOF value of 0.26 h^−1^ for NRR, outpacing that of the traditional noble metal-based NRR electrode in the three-electrode system^7^.

### Catalytic stability

The rectifying contact at the highly coupled interface of Cu and PI also ensures the electrochemical stability of the Cu/PI-300 catalyst for long-term NRR processes. The flat and repeatable i-t curves at −0.4 V vs. RHE in 0.1 mol L^−1^ KOH (Fig. 4c) exhibited negligible attenuation after a 30-h run of standard NRR reactions with the electrolyte renewed every 6 h. The excellent durability of the catalytic active species in the Cu/PI-300 catalyst were also further indicated by the stable NH_3_ yield rates, TOF values (Fig. 4d), and Faradaic efficiencies (Supplementary Fig. 29) for the following four runs of the recycling test. This is especially remarkable as ammonia is known to etch bulk copper to form stable amine-complexes.

## Discussion

In conclusion, herein we present and describe success in designing electron-deficient Cu nanoparticles on semi-conductive PI for application as inexpensive but effective metal catalysts to reduce gaseous dinitrogen under ambient conditions. Importantly, the Mott–Schottky interface contact between the metal and semiconductor tunes the electron density of Cu nanoparticles for preferred adsorption of OH^−^ in basic solution to inhibit the HER process. Simultaneously, electron-deficient Cu nanoparticles remarkably enhance the pre-adsorption of N_2_ molecules for an improved NH_3_ generation yield, as visible even in N_2_-TPD. The present result opens new directions in the search for Mott–Schottky catalysts, using inexpensive and common metals and supports to improve and optimize the specific reaction from impossible to high yield, and resulting in a de novo breakthrough for Cu-catalyzed NRR and a design guideline for other inexpensive metal-based Mott–Schottky catalysts.

## Methods

### Preparation of PI nanoflower

PI nanoflower was synthesized by following a previously reported procedure^28^. In a 250 mL beaker, 1,4-diaminobenzene (1.08 g, 100 mmol) was dissolved into DMF (anhydrous, 60 mL) solution before benzene-1,2,4,5-tetracarboxylic dianhydride (2.18 g, 100 mmol) was added with stirring. The reaction was kept overnight until the viscosity stopped increasing. Then, 30 mL of the solution was transferred into a Teflon-inner autoclave to further polymerization at 180 °C for 10 h. After cooling down, the precipitate in the autoclave was filtrated and washed with DMF and ethanol for several times. The obtained yellowish solid was dried in vacuum overnight and grounded into fine powder. The powder were then heated to 300, 400, or 600 °C at a rate of 5 °C/min and maintained at that temperature for 8 h in a tube furance with N_2_ flow to generate PI-300, PI-400 or PI-600, respectively.

### Preparation of Cu/PI catalyst and Cu/C catalyst

100 mg of PI-300, PI-400, PI-600, or carbon black and 19 mg Cu(NO_3_)_2_·3H_2_O were dispersed into 8 mL of water via sonication and vigorous stir for 2 h. 200 μL 1 M NaOH solution was added to the solution before another two-hour stirring. And then, 0.5 mL of 1 M NaBH_4_ was added dropwise to this suspension. The obtained mixture was separated via centrifugation, washed thoroughly with distilled water and ethanol, then dried in vacuum at 60 °C overnight. The powder was obtained as Cu/PI-300, Cu/PI-400, Cu/PI-600, or Cu/C.

### Preparation of CuO_x_/PI-300 catalyst

100 mg of PI-300 and 19 mg Cu(NO_3_)_2_·3H_2_O were dispersed into 8 mL of water via sonication and vigorous stir for 2 h. 200 μL 1 M NaOH solution was added to the solution before another two-hour stirring. The obtained mixture was separated via centrifugation, washed thoroughly with distilled water, then dried in furnace at 100 °C overnight. The powder was obtained as CuO_x_/PI-300.

### Preparation of Cu/PI/carbon cloth electrodes

20 mg catalyst (Cu/PI-300, Cu/PI-400, or Cu/PI-600), 800 μL of H_2_O, 800 μL of EtOH and 280 μL of 5 wt% Nafion solution in alcohol were mixed and sonicated to generate the catalyst ink. 230/350/470/590/700 μL ink was dropped on the carbon cloth evenly at certain area (1 cm × 1 cm) and then dried at room temperature to afford the Cu/PI/carbon cloth electrodes for electrochemical NRR measurements with the mass loading of 2.50/3.75/5.00/6.25/7.50 mg cm^−2^, respectively.

### Preparation of the reference carbon cloth electrodes

20 mg catalyst (Cu/C or PI-300), 800 μL of H_2_O, 800 μL of EtOH and 280 μL of 5 wt% Nafion solution in alcohol were mixed and sonicated to generate the catalyst ink. 470 μL ink was dropped on the carbon cloth evenly at certain area (1 cm × 1 cm) and then dried at room temperature to afford the reference cloth electrodes for electrochemical NRR measurements with the mass loading of 5.00 mg cm^−2^.

## Supplementary information

Supplementary Information

Source Data

## Data Availability

The data that support the findings of this study are available from the corresponding authors upon request.
