# Supplementary material for: Boosting selective nitrogen reduction to ammonia on electron-deficient copper nanoparticles
Source: Nat Commun. 2019 Sep 26;10:4380. doi: 10.1038/s41467-019-12312-4 (PMC6763479; doi:10.1038/s41467-019-12312-4)
Supplement: Supplementary file 1 — Supplementary Information [file 41467_2019_12312_MOESM1_ESM.pdf]

Boosting Selective N_2 Reduction to NH_3 on Electron-Deficient Cu Nanoparticles

Yun-Xiao Lin[1], Shi-Nan Zhang[1], Zhong-Hua Xue[1], Jun-Jun Zhang[1], Hui Su[1], Tian-Jian Zhao[1], Guang-Yao Zhai[1], Xin-Hao Li[1]*, Markus Antonietti[2] and Jie-Sheng Chen[1]*

[1]*School of Chemistry and Chemical Engineering, Shanghai Jiao Tong University, Shanghai 200240, P. R. China.*

[2]*Department of Colloid Chemistry, Max Planck Institute of Colloids and Interfaces, Wissenschaftspark Golm, Potsdam 14424, Germany.*

** email:* xinhaoli@sjtu.edu.cn (XHL) chemcj@sjtu.edu.cn (JSC)

Materials and Methods:

1. Materials

All reagents and solvents were purchased from Adamas-beta and used without further purification. Carbon black (Vulcan XC 72) was purchased from Cabot and graphitized at 900 °C for two hours in N_2 gas. Carbon cloth (W0S1002) were purchased from CeTech, Taiwan, China. F-doped SnO_2 (FTO) transparent substrate electrodes (sodalime, sheet resistance: 14 Ω sq^{-1}) were purchased from Nippon Sheet Glass, Japan. All electrodes were cut into small pieces of 2 cm × 1 cm, washed for three times with distilled water, ethanol and acetone successively and dried under air flow for further use.

2. Characterization

Fourier transform infrared (FT-IR) spectra were collected on a Spectrum 100 spectrometer. The sample was ground with spectrographically KBr and tabletted as a transparent slice. UV-Vis spectra were recorded on a Shimadzu UV-2450 UV-Vis spectrophotometer. Powder X-ray Diffraction (PXRD) data were collected on a Bruker D8 Advance X-ray diffractometer with Cu-Kα radiation (λ = 1.5418 Å) with a scan rate of 6° min^{-1}. Solid state nuclear magnetic resonance (SSNMR) measurements were performed on a Bruker Avance Ⅲ 400 MHz system. Scanning electron microscopy (SEM) images were acquired on a FEI Nova NanoSEM 2300 microscope. Transmission electron microscopy (TEM) images were acquired on JEM-2100 microscope at an electron acceleration voltage of 200 kV. Samples were prepared by clamp a piece of coated carbon fiber between two Cu grids. High-angle annular dark-field (HAADF), high-resolution TEM (HRTEM) and Energy Dispersive X-Ray (EDX) images were acquired on a TALOS F200X microscope. The X-ray photoelectron spectroscopy (XPS) and ultraviolet photoelectron spectra

(UPS) measurements were conducted on a Kratos Axis Ultra DLD spectrometer using a monochromated Al Kα radiation. Inductively coupled plasma (ICP) optical emission measurements were conducted on an iCAP7600 spectrometer. Temperature programmed desorption (TPD) tests were carried on a Micrometritics AutoChem Ⅱ chemisorption analyzer. Low-pressure gas adsorption measurements were performed on a Nova 2200e surface area & pore size analyzer. Samples were degassed under dynamic vacuum for 12 h at 100 ^{o}C prior to each measurement. N_2 isotherms were measured using a liquid nitrogen bath (77 K).

3. Electrochemical measurements

Electrochemical NRR performances were investigated using a CHI 730 C electrochemistry workstation (CH Instruments, Inc., Shanghai) in a 150 mL five-necked flask based on a standard three-electrode system. The three-electrode electrochemical cell was made up of a Cu/PI/carbon cloth electrode, a graphite rod and a saturated calomel electrode (SCE), which were served as working electrode, counter electrode, and reference electrode, respectively. The NRR tests were performed in N_2 saturated 0.1 mol L^{-1} KOH solution (90 mL). The electrolyte was stirred and bubbled with N_2 gas for 30 min before the tests. During the tests, the N_2 flow was continuously inputted using properly positioned spargers so that the whole cathode was hit by the gas bubbles where N_2 and water (H_2O) combine with electrons to form hydroxide (OH^-) and the N_2 reduction product. For comparison, potentiostatic tests in Ar saturated 0.1 mol L^{-1} KOH solution were also conducted in this work. Linear sweep voltammograms (LSV) measurements were conducted with a scan rate of 10 mV s^{-1}. All the LSV curves are the steady-state ones after several cycles. The long-term NRR tests were performed using chronoamperometric measurements. An empty glass tube was set at the end of the flask. After NRR reaction, the liquid in glass tube was poured back into the flask for following ammonia determination. All potentials were described versus the

reversible hydrogen electrode (RHE) via the following equation: $E_{RHE} = E_{SCE} + 0.059\ V \times pH + 0.241\ V$. Cyclic voltammogram (CV) measurements were conducted by potential cycling in Ar saturated 0.1 mol L^{-1} KOH solution to evaluate the corresponding HER performance. The working electrode was the rotating disk electrode (RDE) with a mass loading of Cu/PI-300 at 1 mg cm^{-2}. The rotate speed was around 1000 rpm and the curves were iR corrected.

The CV curves were converted as overpotential vs log current (log j) to get Tafel plots. Tafel slope was obtained by fitting the Tafel plots (the linear portion) to the Tafel equation ($\eta = b\log(j) + a$). Mott–Schottky plots were performed in a typical three-electrode system in the 0.1 mol L^{-1} PBS solution on an electrochemical workstation (CHI 660C) by using the Impedance-Potential technique. The Cu/PI/FTO electrodes were prepared using the same method referred to the Cu/PI/ carbon cloth electrode and used as the working electrode. A graphite rod was used as the counter electrode and SCE was used as the reference electrode. All measurements were performed at room temperature.

4. Determination of ammonia

Colorimetric method

The quantity of NH_3 formation was determined via a reported colorimetric method[S1] using Nessler's reagent. Calibration curve (Supplementary Figure 13b) was plotted according to the following method: Firstly, a series of reference solutions were prepared by adding suitable volumes of the ammonia working 0.1 M KOH solution in colorimetric tubes. Then, the solution was made up to the mark (10 mL) with 0.1 M KOH solution before adding 1 mL of 0.2 M potassium sodium tartrate ($KNaC_4H_4O_6$) solution to each of the tubes and mix thoroughly; Next, 1 mL of Nessler's reagent was added to each of the tubes and the solution was standing for 20 minutes for color development; Finally, the absorbance of the solutions was measured at 425 nm

using a 10 mm glass cuvette.

Ion chromatography method

After NRR test, 10 mL obtained NH_3 solution in 0.1 M KOH was adjusted pH to 3 with 1.01 mL 1 M HCl and filtered through a nylon membrane filter (0.45 mm) before analysis by Ion Chromatography (Dionex 1500i with an AS 4 ASC column; Sunnyvale, USA). The NH_4^+ peak was observed at 7.05 min. Calibration curve (Supplementary Figure 14b) was built to quantify the ammonium ion in the solution.

$^{15}N_2$ isotope labeling experiments

99% $^{15}N_2$ was used as the feeding gas to perform the isotopic labeling NRR experiment in order to clarify the source of ammonia. The electrochemical reactor was sealed, degassed and filled with Ar for three times, then refilled with $^{15}N_2$ gas. 20 mL $^{15}N_2$ was injected to the system every 10 min during the test. After NRR for 6 h, the pH value of obtained solution was adjusted to 7 with 0.5 M H_2SO_4 and $^{15}NH^{4+}$ was identified using ^1H NMR spectroscopy on the obtained solutions (Supplementary Figure 15). $^{14}N_2$ experiment was also performed in the same condition for comparison.

5. Faraday efficiency, generation rate and turnover frequency

The Faradaic efficiencies for NRR were defined as the quantity of electric charge used for synthesizing ammonia divided by the total charge passed through the electrodes during the electrolysis. The Faraday efficiency (FE) can be calculated as FE = nN × 96485/(It), where N is the electron number for the NRR (here, N is 3); n represents the mole of NH_3; I represents the current (A); and t represents time (s). The generation rate (GR) of ammonia was calculated using the following equation: GR = 17n/(tA), where n represents nNH_3; t represents time (h); and A is

the effective area of the electrode (cm^2). The turnover frequency (TOF) can be calculated by the equation: TOF = n/(tn'), where n represents the mole of NH_3; t represents time (h); and n' represents the mole of Cu on the electrode.

6. Theoretical calculations

All theoretical calculations were performed using density functional theory (DFT), as implemented in the DMol3 program based on Materials Studio 8.0. The electronic exchange-correlation energy was described by the generalized gradient approximation (GGA) method with spin polarized Perdew-Burke-Ernzerhof (PBE) functional.[S2] Valence orbitals were described with the double numerical plus polarization (DNP) basis.[S3] The Cu (111) surfaces in the experiment were modelled as a Cu model by cutting a 3×3×3 repetitive rhomb Cu cluster (i.e. pristine Cu).[S4] A specific charge of e^- was reduced by each of Cu atom to form the electron-deficient Cu (Cu-0.04e^- and Cu-0.04e^-) models.[S5] The k-space integration was sampled using a 6×6×1 Monkhorst-Pack grid.

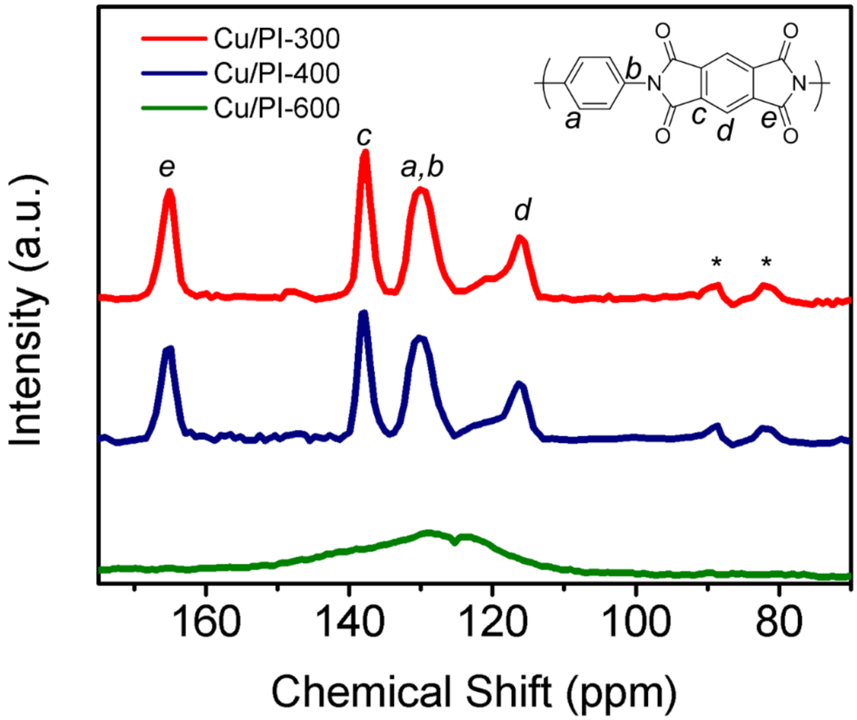

Supplementary Figure 1. SSNMR spectra of Cu/PI catalysts. Asterisks (*) indicated the peaks arising from spinning side bands. The peaks of Cu/PI-600 were broad and weak, rather speaking for a carbonized polymer framework.

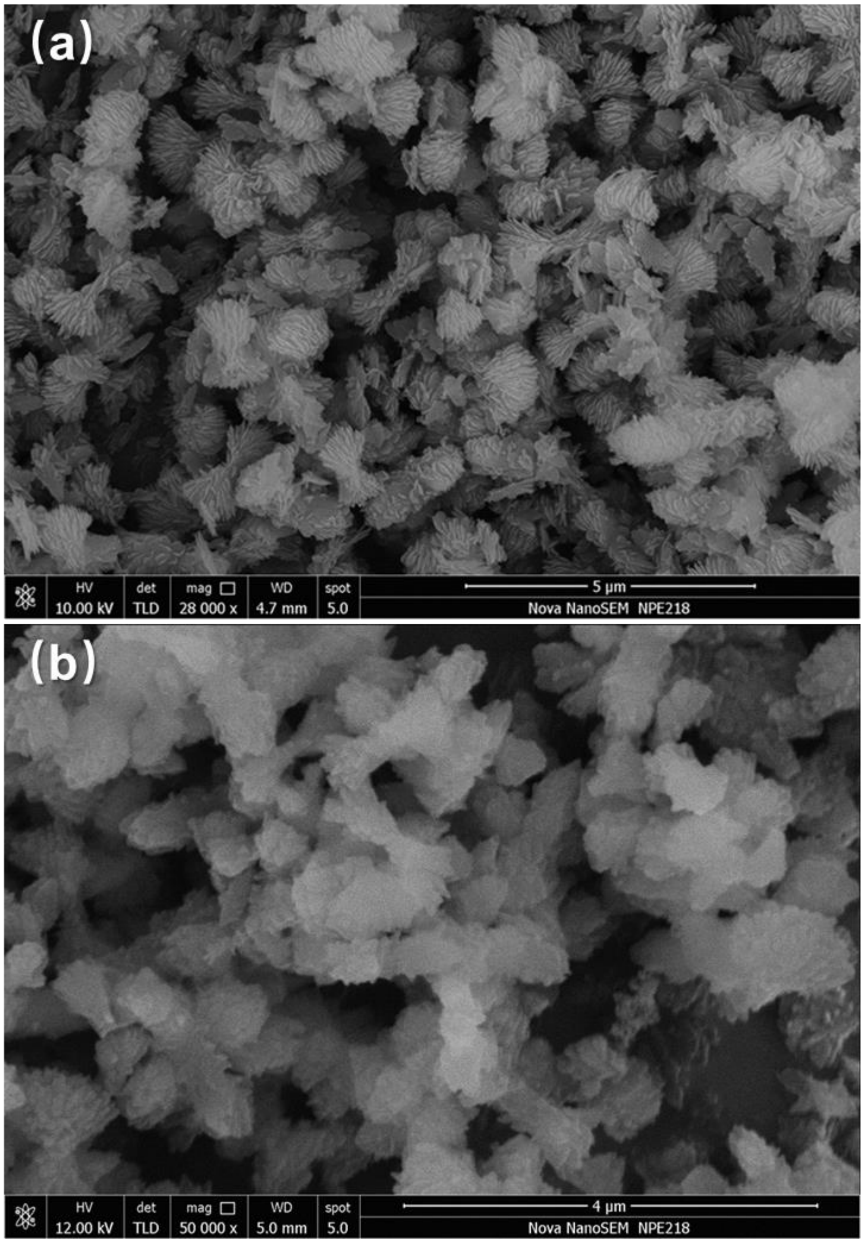

Supplementary Figure 2. SEM images of (a) PI-300 and (b) Cu/PI-300 samples. The morphology of PI support remained nearly the same after the introduction of Cu nanoparticles.

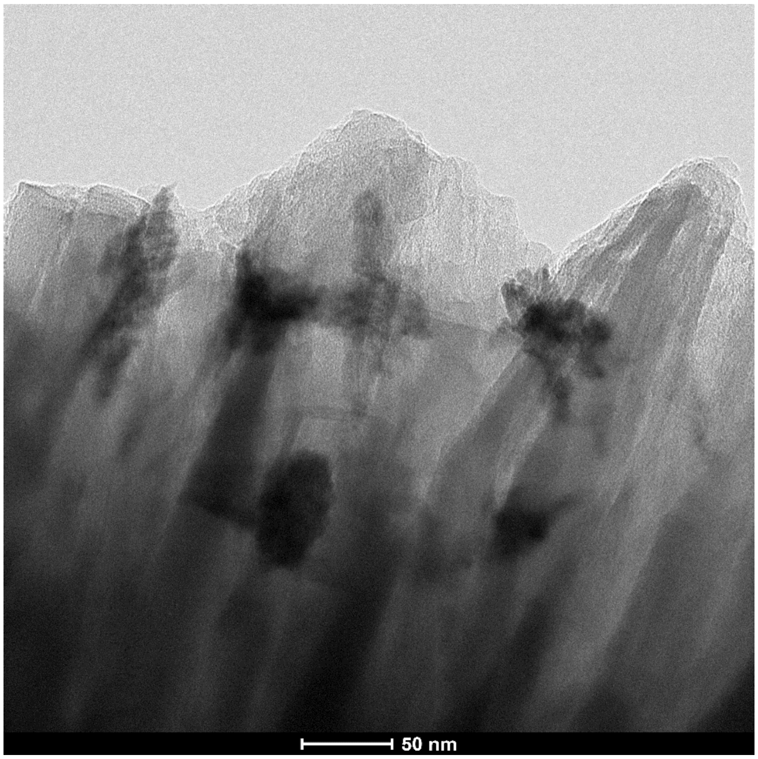

Supplementary Figure 3. TEM image of Cu/PI-300 catalyst.

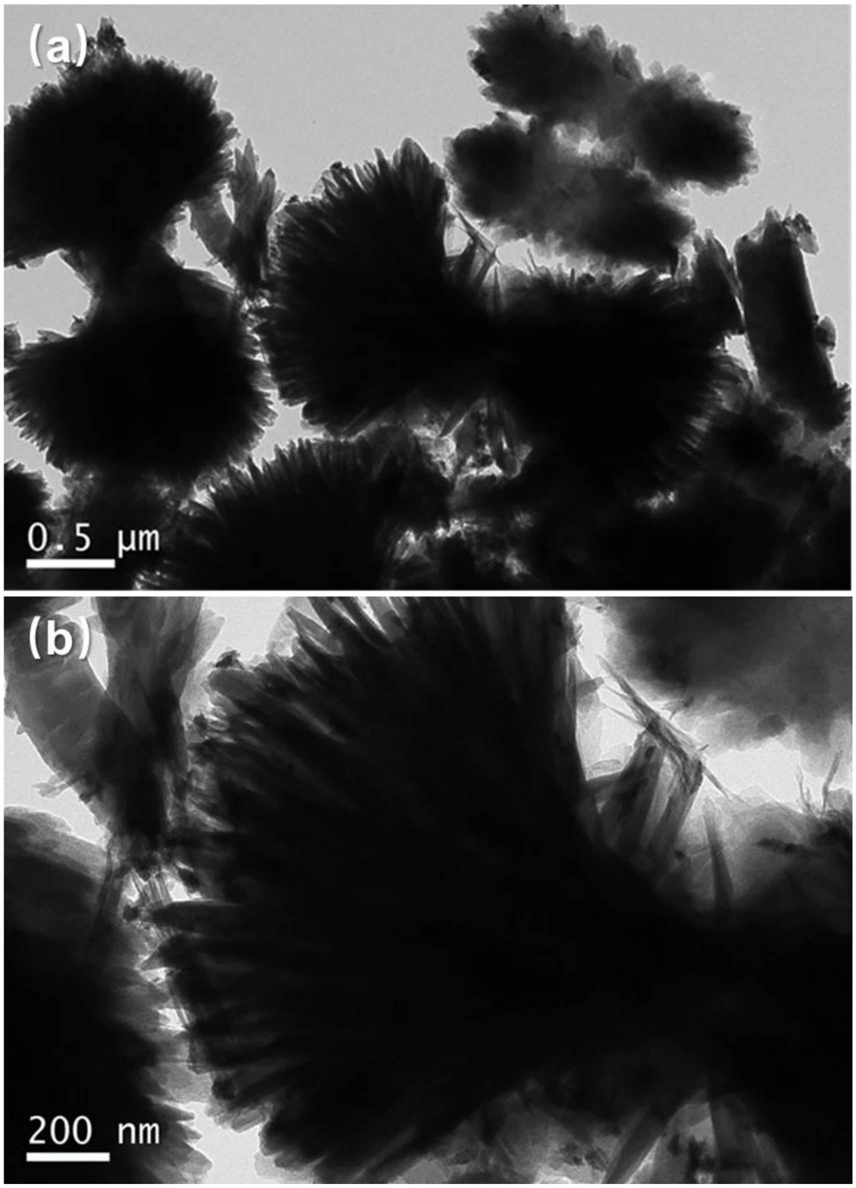

Supplementary Figure 4. TEM images of Cu/PI-400 catalyst.

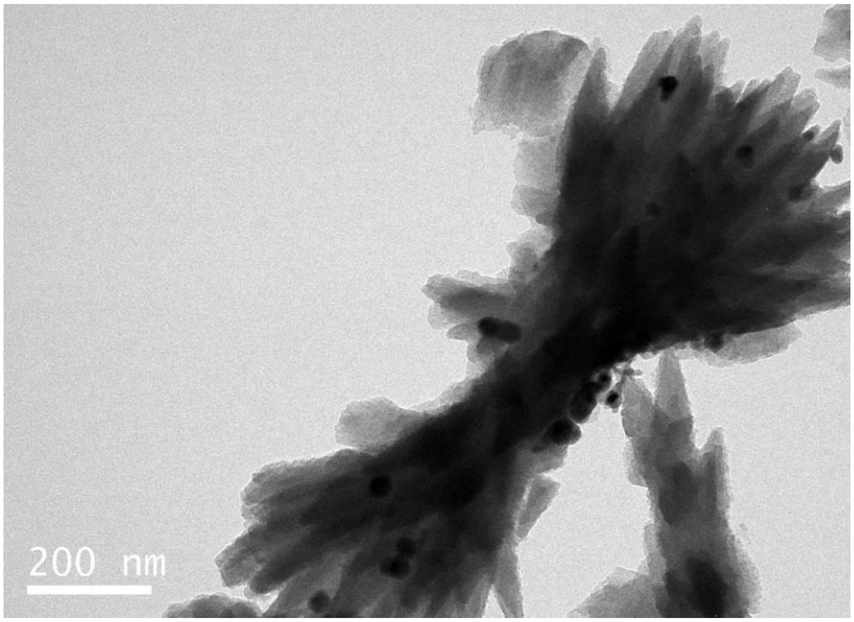

Supplementary Figure 5. TEM image of Cu/PI-600 catalyst.

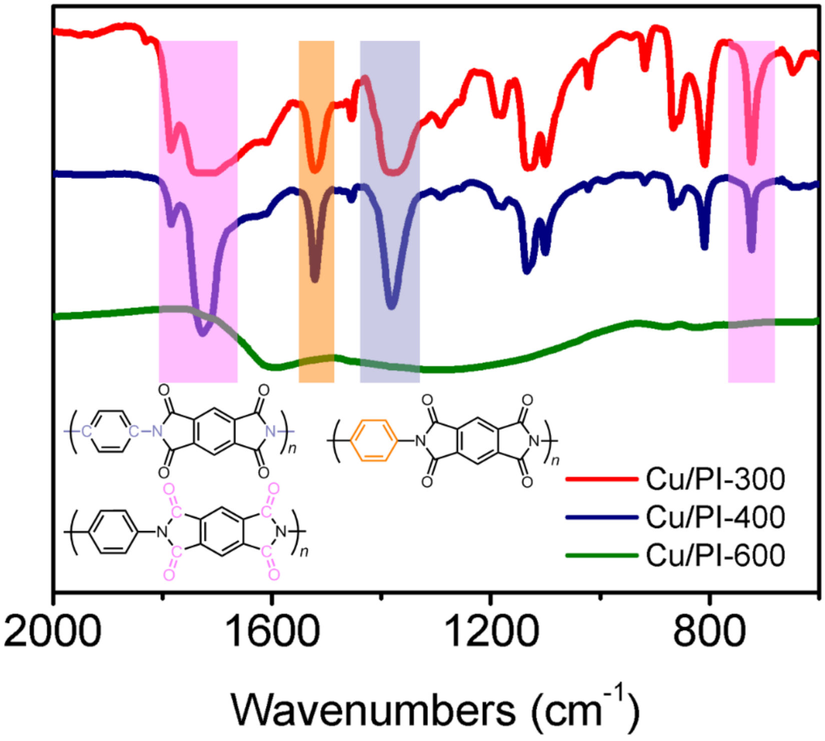

Supplementary Figure 6. FT-IR spectra of Cu/PI catalysts. IR band assignments of Cu/PI-300 and Cu/PI-400 were agreed with the reported data[S6] of PI (imide C=O, ν_{as}: 1783 cm^{-1}, imide C=O, ν_{s}: 1727 cm^{-1}, imide C-N, ν: 1381 cm^{-1}, imide C=O, δ: 722 cm^{-1}, pPDA benzene: 1522 cm^{-1}), indicating the sSupplementary Table tructure of PI support after the deposition of Cu components. The spectrum of Cu/PI-600 suggested the carbonization of the PI support at a high synthetic temperature.

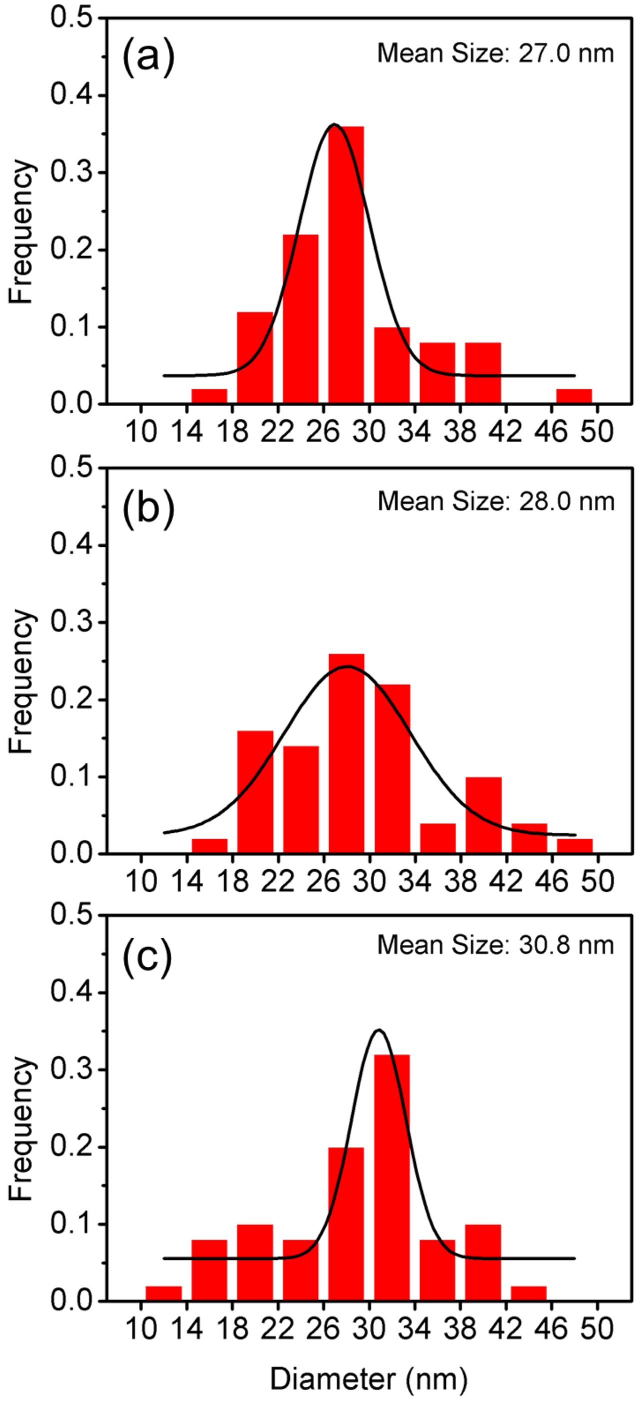

Supplementary Figure 7. The Cu nanoparticle size distribution of Cu/PI-300 (a), Cu/PI-400 (b) and Cu/PI-600 (c) according to TEM images. All samples had similar Cu nanoparticle size distributions around 30 nm, suggesting the negligible effect of particle size on the NRR activity.

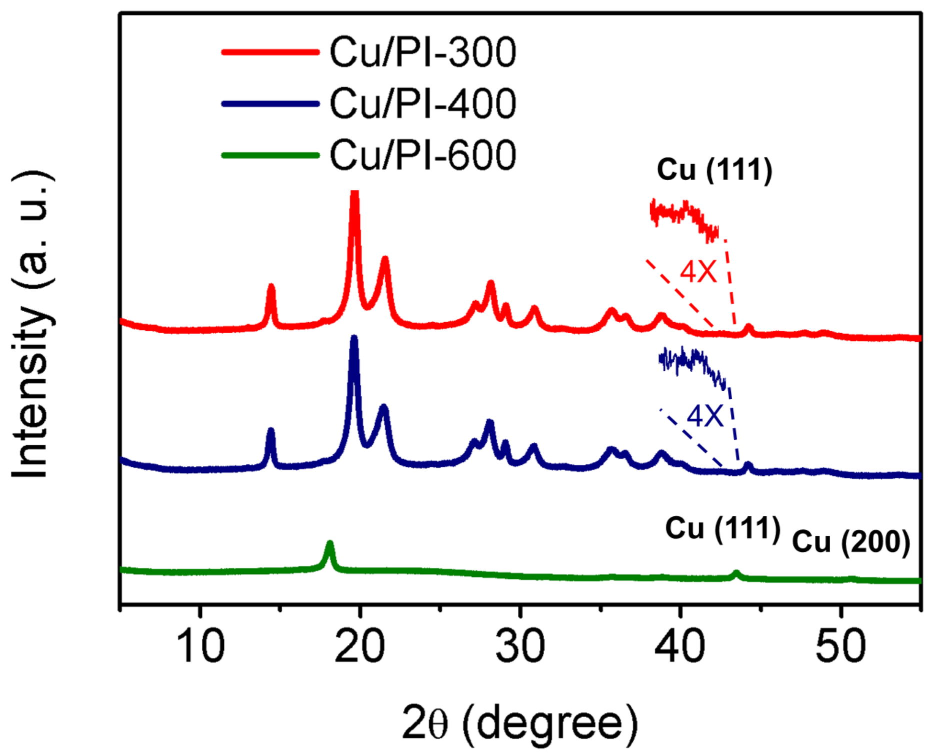

Supplementary Figure 8. PXRD patterns of Cu/PI catalysts. The XRD peaks of Cu/PI-300 and Cu/PI-400 samples matched well with those of PI materials in the literature.[S7] Typical XRD signals of Cu (111) were observed in all three samples. The amorphous feature of the support of Cu/PI-600 rather suggested the formation of organic carbon with a low graphitization degree during the carbonization process at 600 ^{o}C.

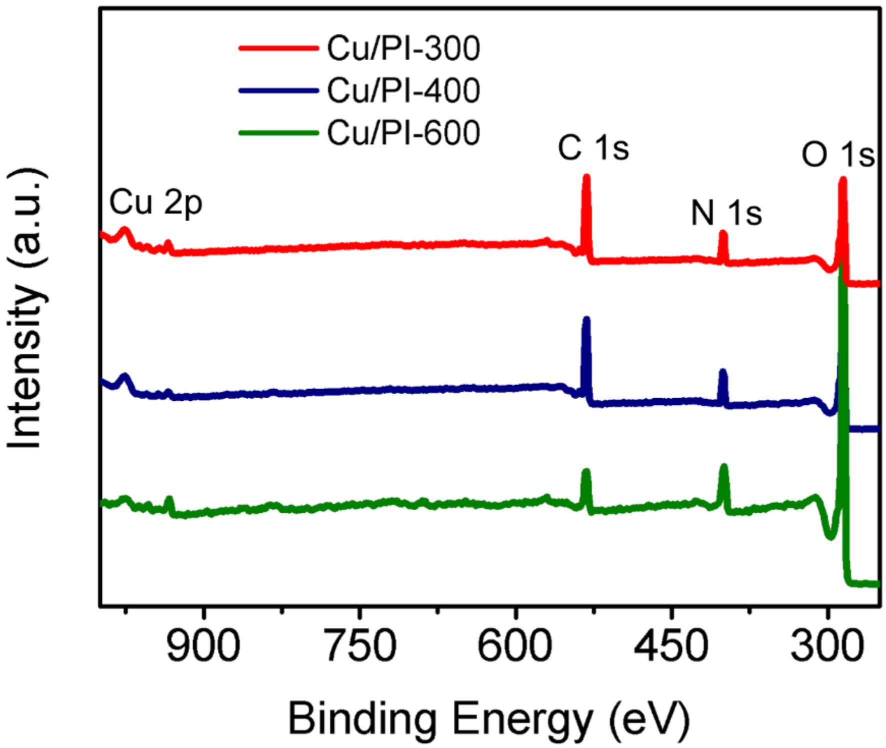

Supplementary Figure 9. X-ray photoelectron spectroscopy (XPS) survey spectra of Cu/PI catalysts.

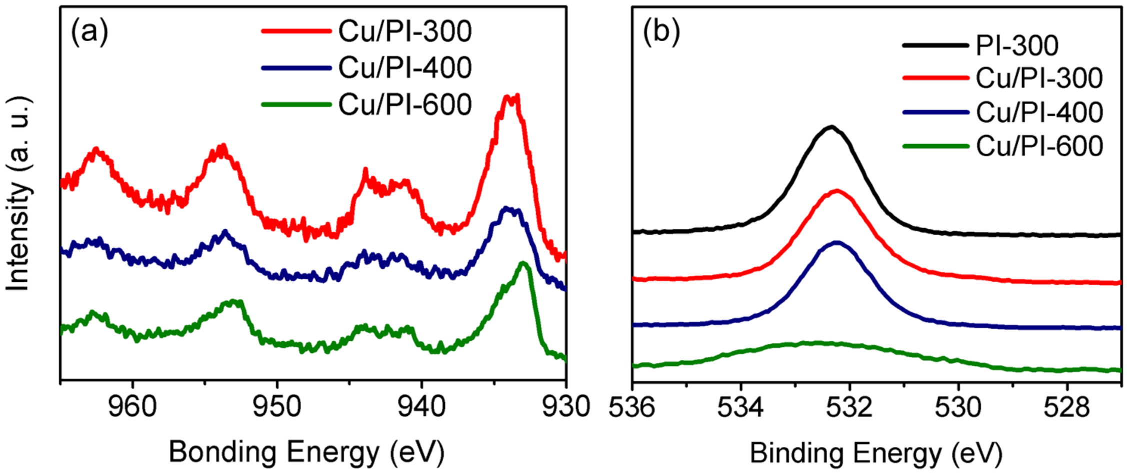

Supplementary Figure 10. High resolution XPS spectra of (a) Cu and (b) O of Cu/PI catalysts. The Cu 2p peak shape in Cu/PI-300, Cu//PI-400 and Cu/PI-600 were mainly consistent, which demonstrated the metallic state of supported Cu species in all control samples. The O 1s signals in Cu/PI-300, Cu//PI-400 and Cu/PI-600 were all attributed to the carboxyl O of polyimide and no other copper oxides or hydroxides peaks can be observed, which exclude the presence of lattice oxygen in Cu nanoparticles. For the Cu/PI-600, the peak was board due to the carbonization of the structure.

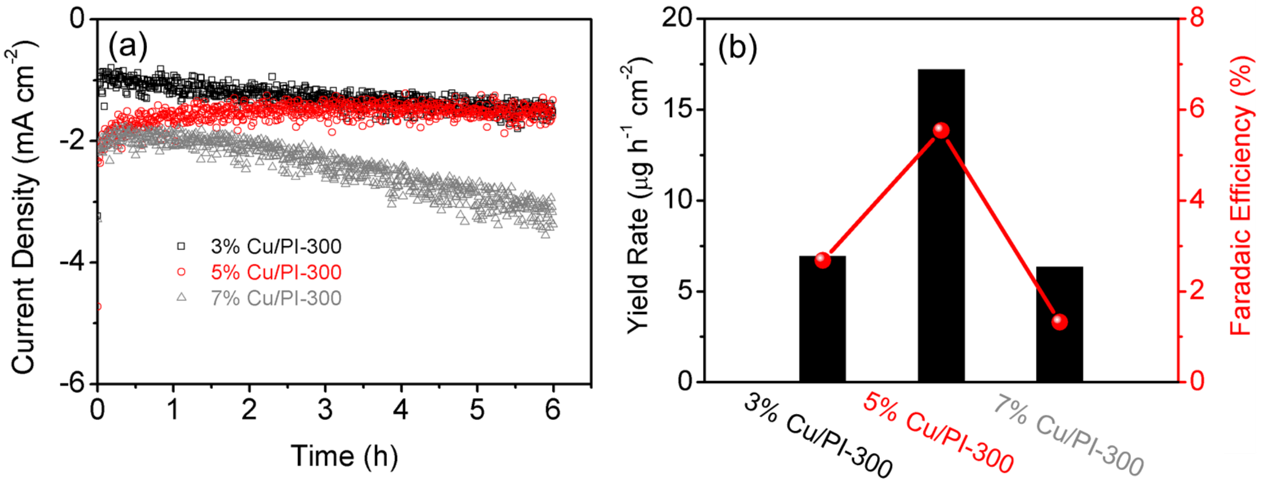

Supplementary Figure 11. (a) Chronoamperometry curves of Cu/PI-300 electrodes with 3%, 5% and 7% Cu content (catalyst loading: 5 mg cm^{-2}) at a potential of -0.4 V vs. RHE for 6 h in N_2-saturated KOH (0.1 M). (b) The corresponding Faradaic efficiencies (spheres) and NH_3 yield rates (bars) of Cu/PI-300 with 3%, 5% and 7% Cu content. The Cu content of Cu/PI-300 electrodes were optimized to be 5% in the following experiments.

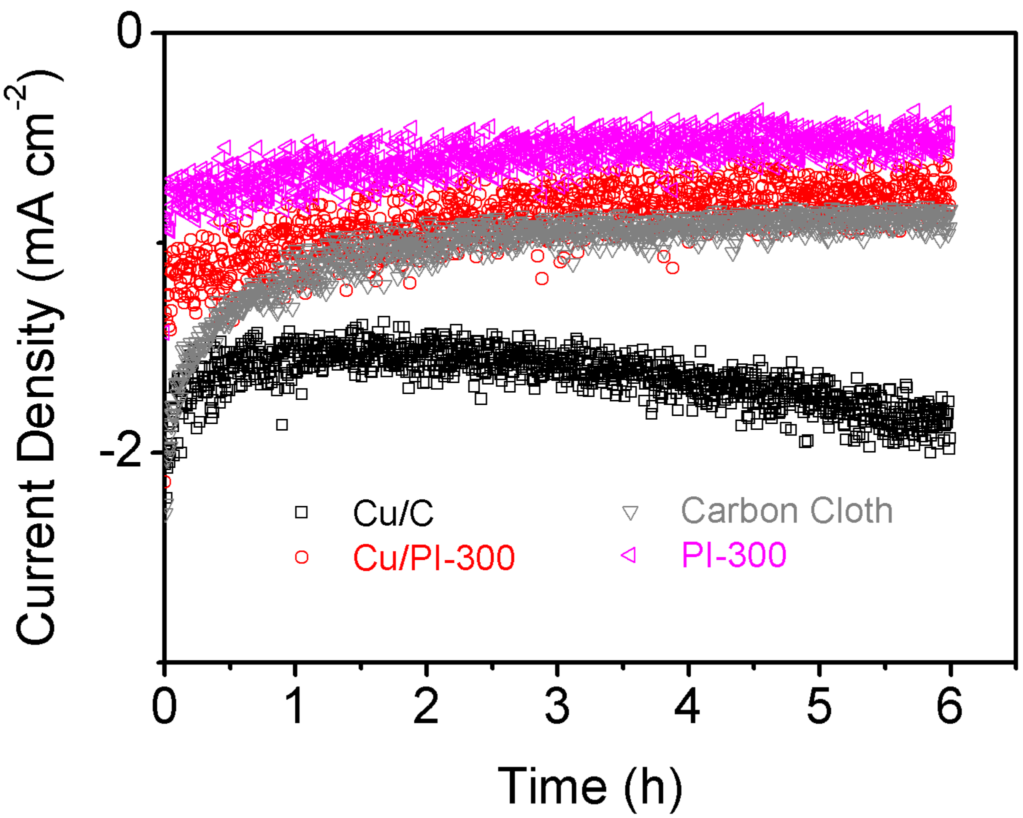

Supplementary Figure 12. Chronoamperometry curves of Cu/PI-300, PI-300 and Cu/C electrodes with a fixed catalyst loading (5 mg cm^{-2}) at a potential of -0.3 V vs. RHE for 6 h in N_2-saturated KOH (0.1 M). The stability of bare carbon cloth was also tested for comparison.

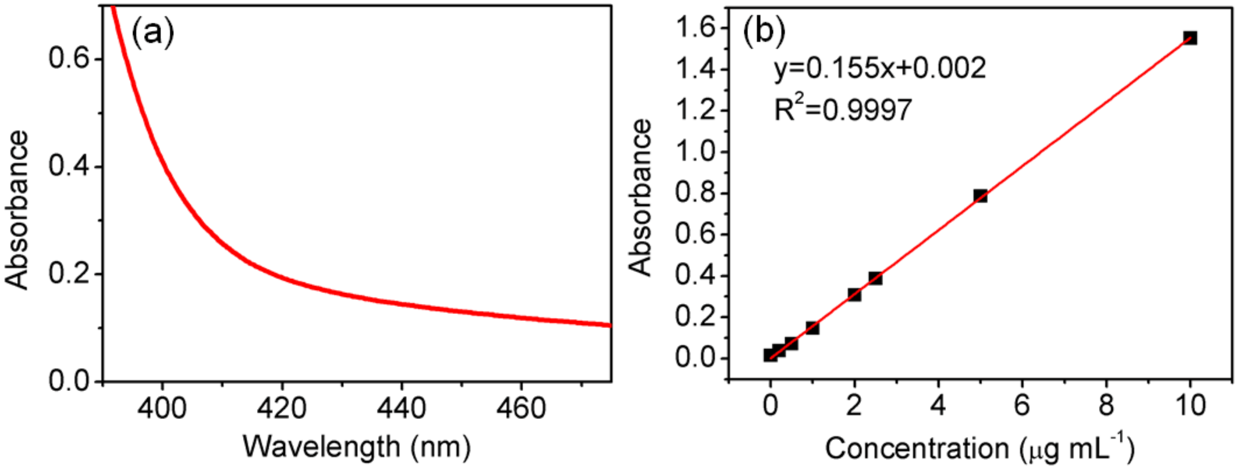

Supplementary Figure 13. The UV-Vis spectrum (a) and corresponding calibration curve (b) for evaluating the NRR performance of various catalysts by using the Nessler's reagent. The line showed a linear fit (y = ax + b) of N = 3 independent calibration curves. The test spectrum was obtained and the NH_3 absorbance was observed at 425 nm. The yield rate of ammonia for the best-in-class electrode (Cu/PI-300) under optimized condition was calculated as 17.2 μg h^{-1} cm^{-2}.

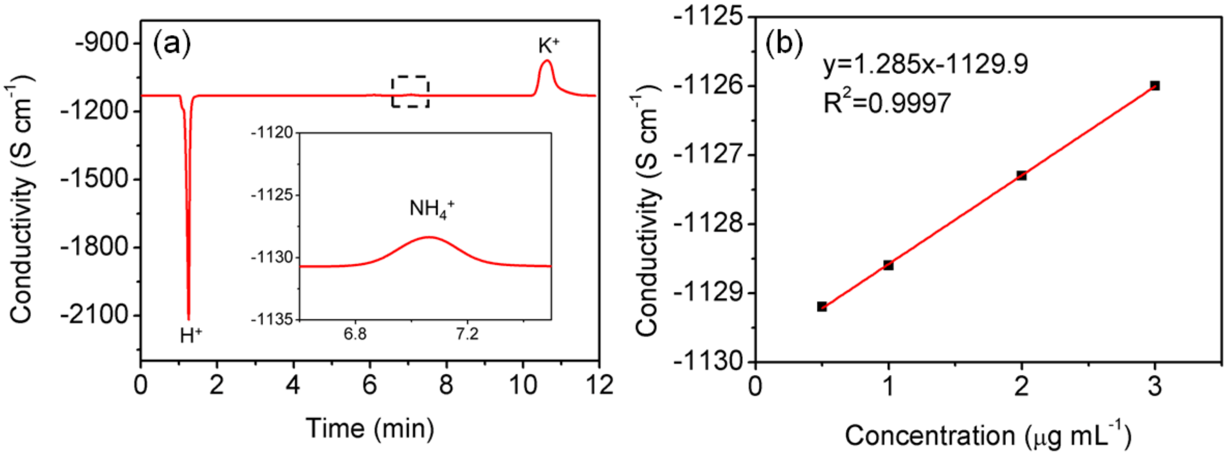

Supplementary Figure 14. The ion chromatography spectrum (a) and corresponding calibration curve (b) for the evaluation of NRR performance of various catalysts. The pH value of 10 mL of 0.1 M KOH electrolytes containing a certain amount of ammonia was adjusted to 3 by using 1.01 mL of 1 M HCl before tests. The line showed a linear fit (y = ax + b) of N = 3 independent calibration curves. The test spectrum was obtained and the NH_4^+ peak was observed at 7.05 min. The yield rate of ammonia for the best-in-class electrode (Cu/PI-300) under optimized condition was calculated as 20.1 μg h^{-1} cm^{-2}, which was closely concordant with the colorimetric result (17.2 μg h^{-1} cm^{-2}) as depicted in Supplementary Figure 13. Generally speaking, the slightly different values from the ion chromatography spectra were mainly induced by the possible conductivity background from K^+ and H^+ cations with very high concentrations as described in previous work[S8-9].

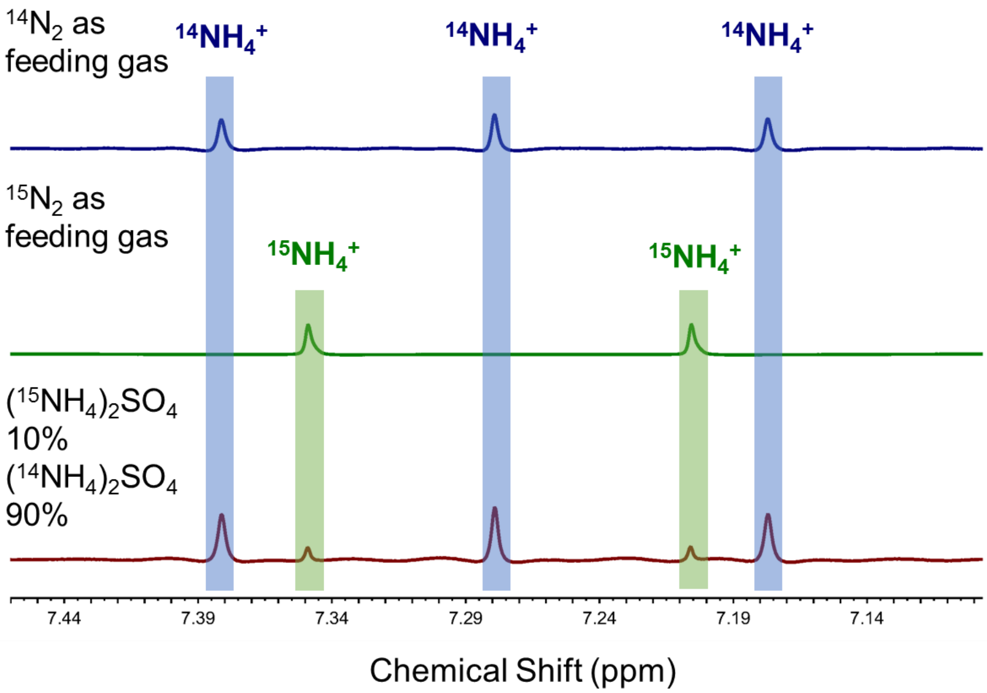

Supplementary Figure 15. ^1H NMR spectra (500 MHz, DMSO-d_6) of both $^{14}NH_4^+$ and $^{15}NH_4^+$ generated from the NRR reaction using $^{14}N_2$ and $^{15}N_2$ as feeding gas, respectively. The NMR spectrum of the mixture of [$(^{15}NH_4)_2SO_4$ 10%, $(^{14}NH_4)_2SO_4$ 90%] was also given as a reference.

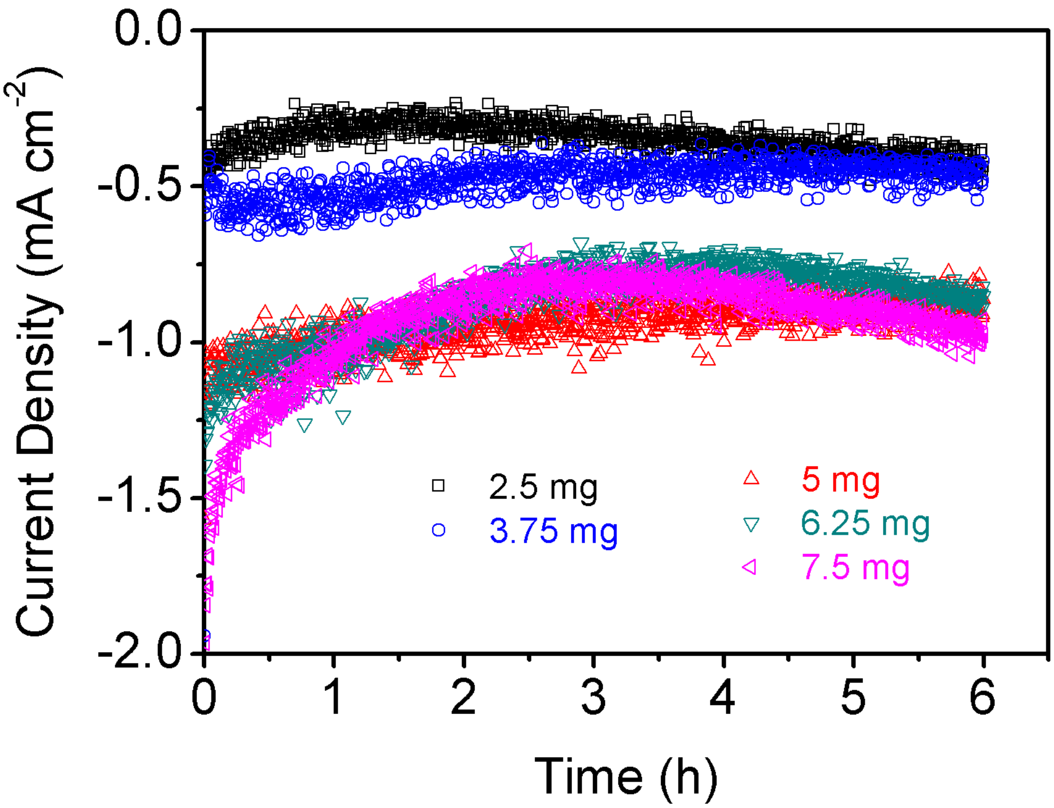

Supplementary Figure 16. Chronoamperometry curves of Cu/PI-300 electrodes with varied catalyst loadings at a potential of -0.3 V vs. RHE for 6 h in N_2-saturated KOH (0.1 M)**.**

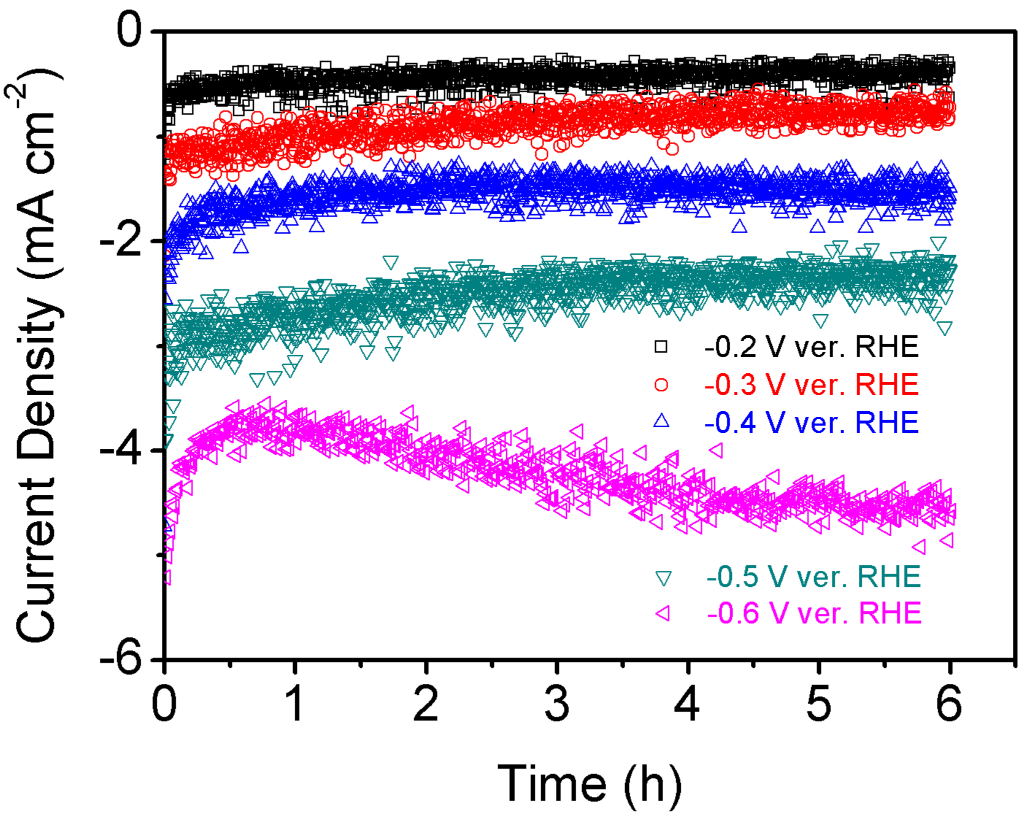

Supplementary Figure 17. Chronoamperometry curves of Cu/PI-300 electrodes with a fixed catalyst loading (5 mg cm^{-2}) at various potentials vs. RHE for 6 h in N_2-saturated KOH (0.1 M).

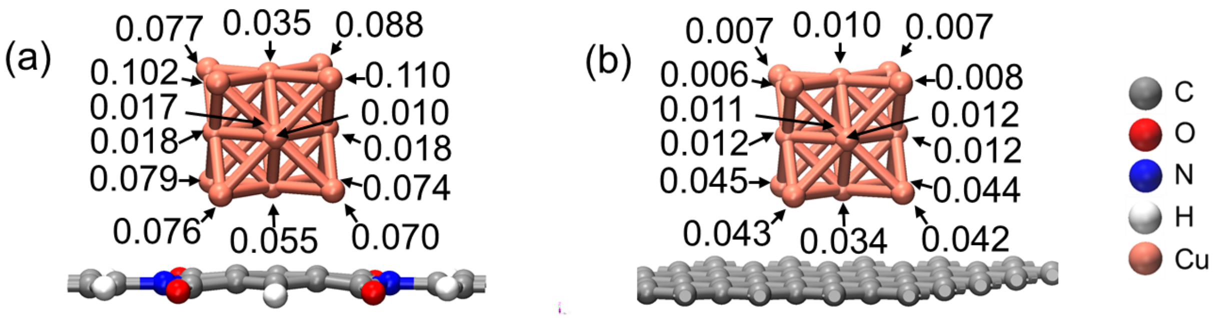

Supplementary Figure 18. Hirshfeld charge for each Cu atom on the surface of (a) polyimide and (b) carbon support. The significant difference in Hirshfeld charge indicated that the PI as a semiconductor support can attract more electrons from the Cu cluster (0.06 for each Cu atom) than that by carbon support (0.02 for each Cu atom) in Cu/C model.

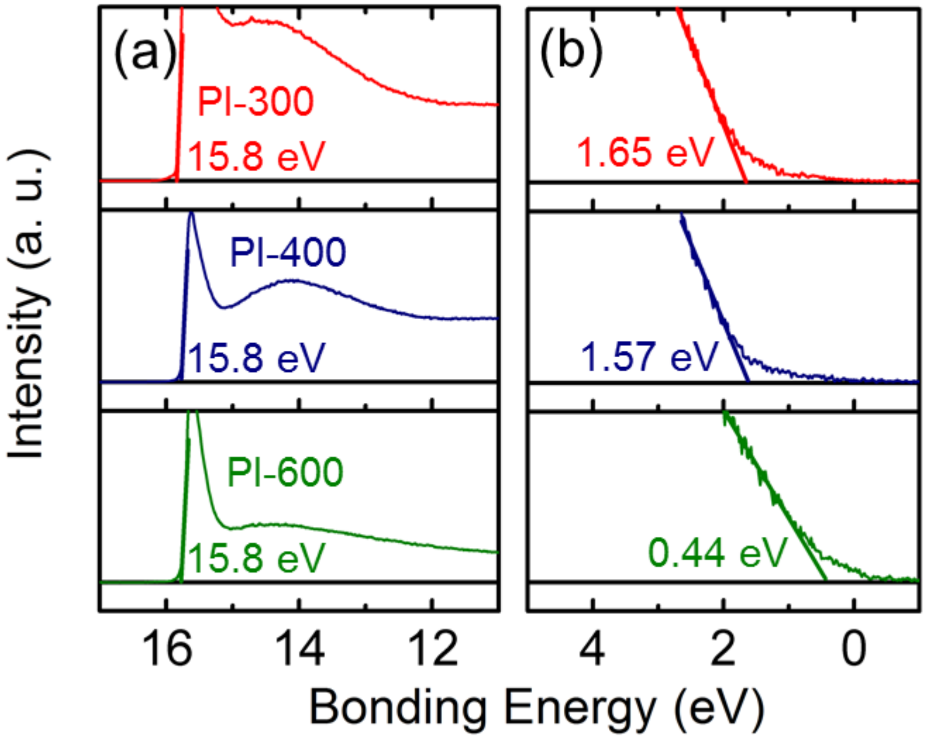

Supplementary Figure 19. (a) UPS spectra in the cutoff (E_{cutoff}) and (b) onset (E_i) energy regions of various PI samples. The ionization energy values of PI-300, PI-400 and PI-600 were determined by the equation of $\Phi = 21.21$ eV – ($E_{cutoff} - E_i$) as 7.06, 6.98, 5.85 eV, respectively.

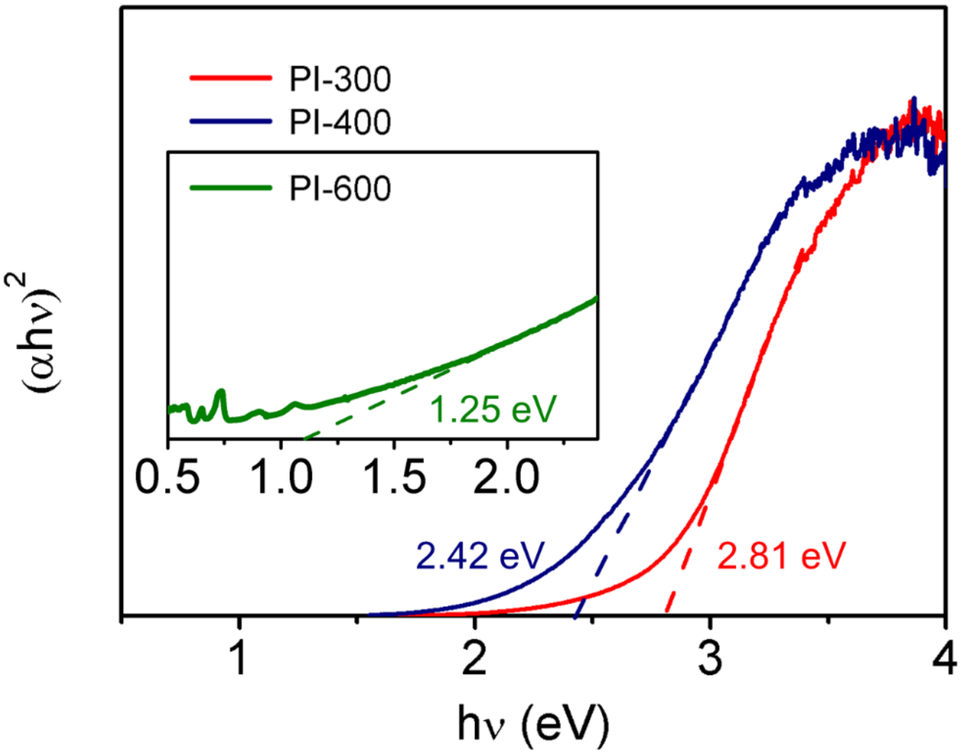

Supplementary Figure 20. The Tauc curves of PI supports according to the UV-vis spectra. As a result, the band gaps (E_g) of PI-300, PI-400 and PI-600 were estimated to be 2.81, 2.42 and 1.25 eV, respectively.

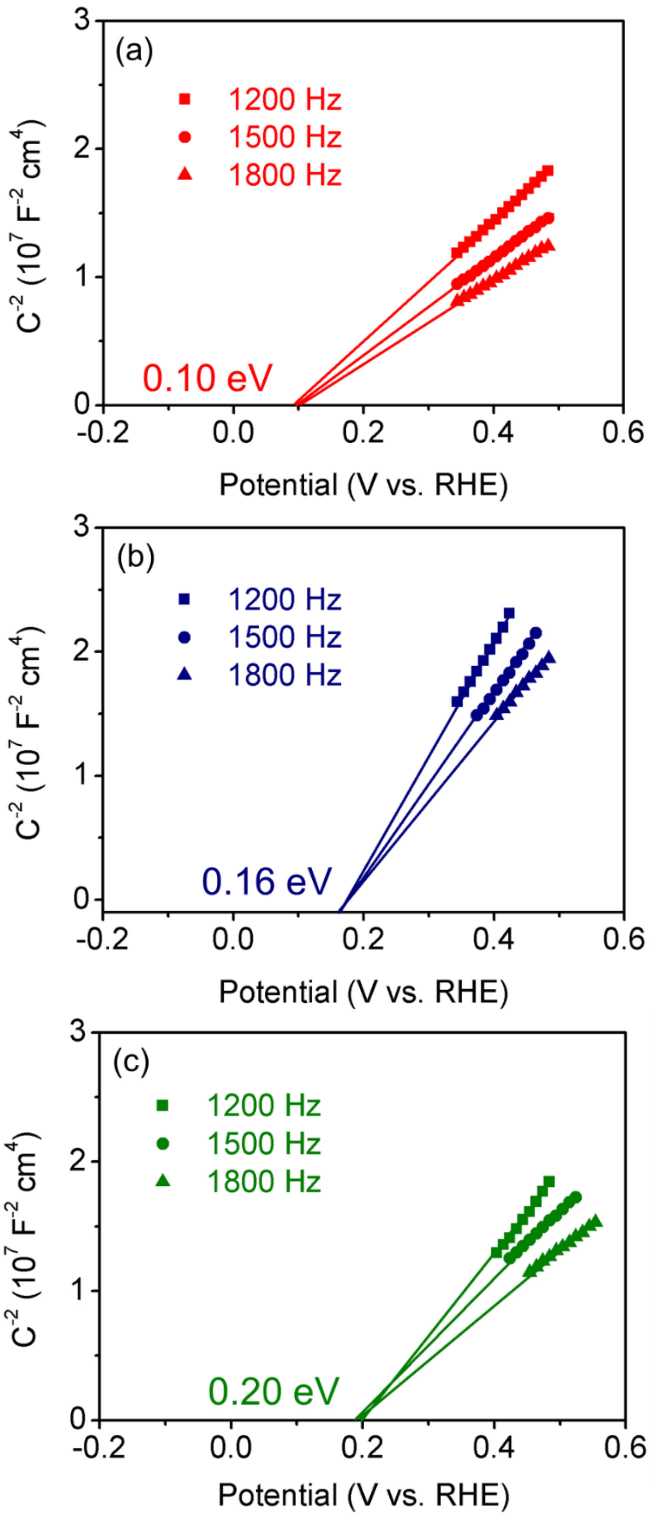

Supplementary Figure 21. Mott-Schottky plots of (a) PI-300, (b) PI-400 and (c) PI-600 at various frequencies, demonstrating the conduction band potentials as 0.10, 0.16 and 0.20 eV vs RHE, respectively.

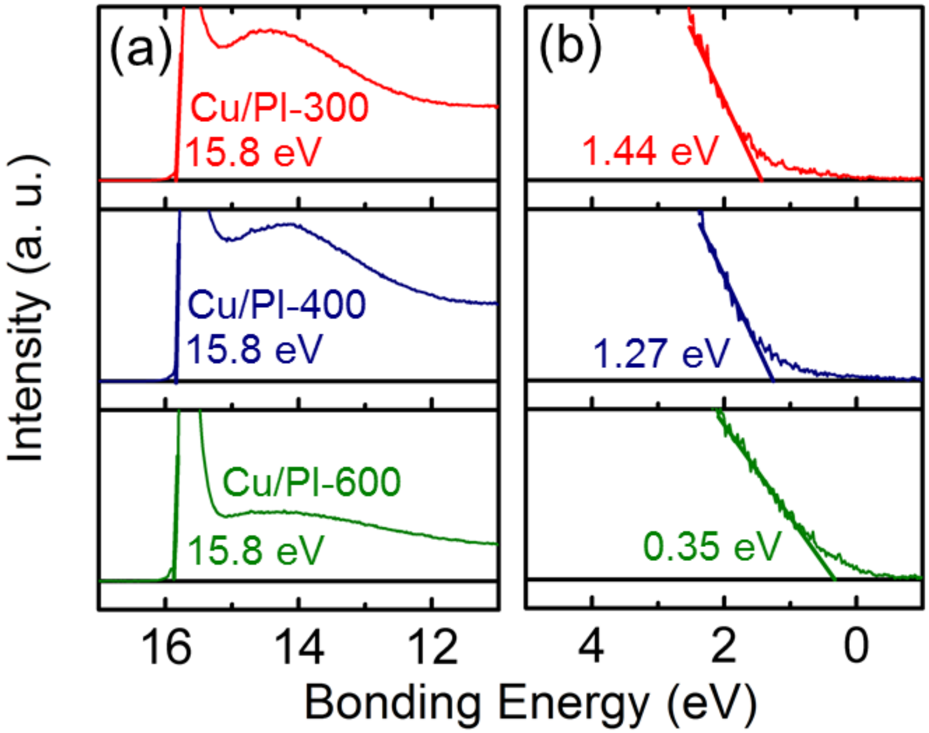

Supplementary Figure 22. (a) UPS spectra in the cutoff (E_{cutoff}) and (b) onset (E_i) energy regions of Cu/PI catalysts. The ionization energy values of Cu/PI-300, Cu/PI-400 and Cu/PI-600 were determined by the equation of $\Phi = 21.21$ eV – ($E_{cutoff} - E_i$) as 6.85, 6.68, 5.76 eV, respectively.

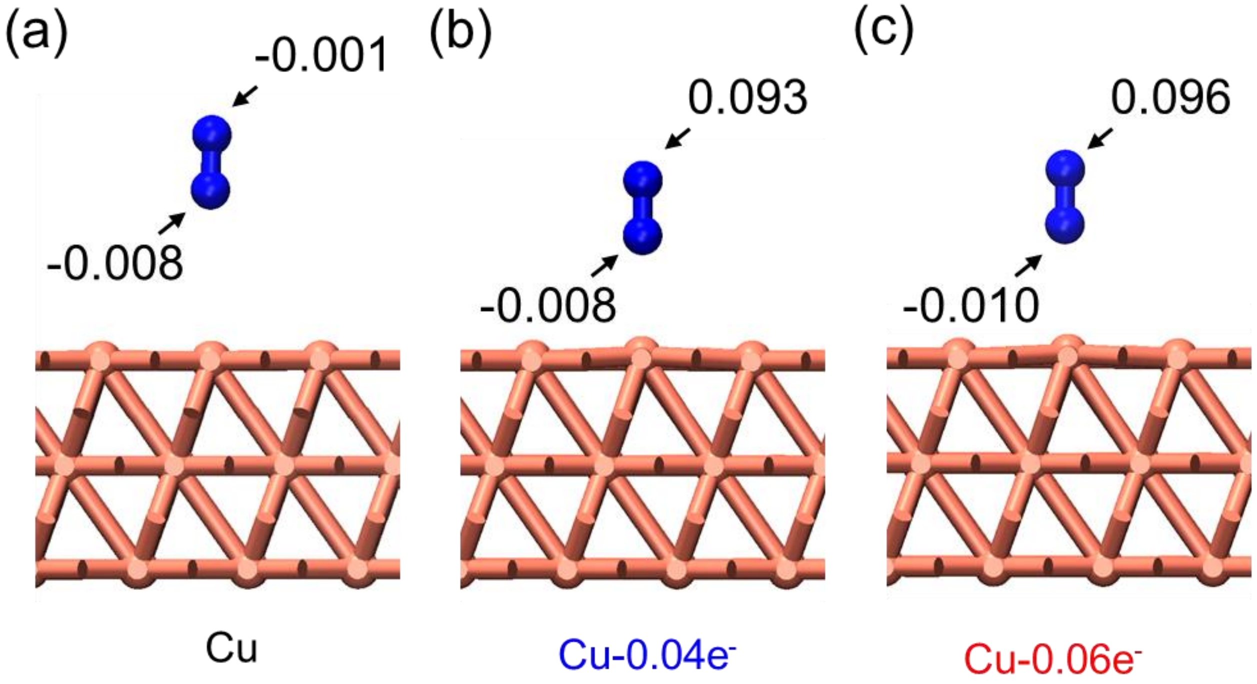

Supplementary Figure 23. Hirshfeld charge for each N atom on the surface of (a) pristine Cu, (b) Cu-0.04e$^-$ and (c) Cu-0.06e$^-$. The significant difference in Hirshfeld charge of the top N atom on each support directly certified that N_2 molecule on electron-deficient Cu was highly polarized and activated.

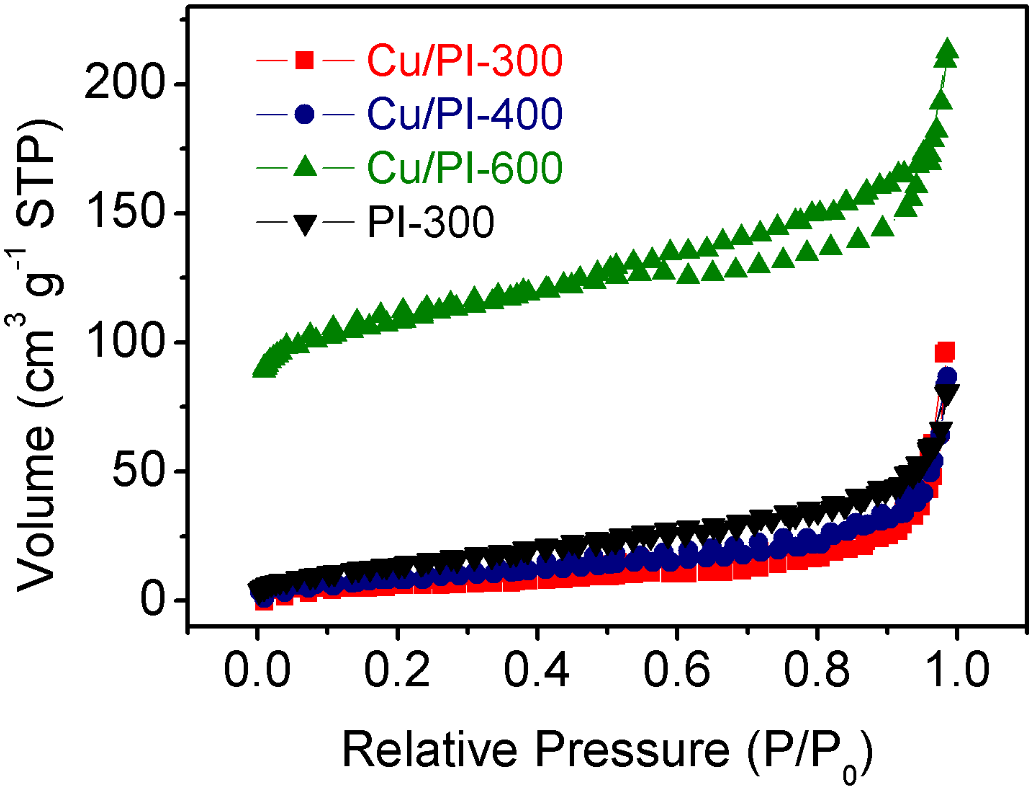

Supplementary Figure 24. The N_2 adsorption isotherm curves of Cu/PI catalysts and bare PI-300 at 77K. The Brunauer-Emmett-Teller (BET) surface areas were estimated as 36, 42, 42 and 336 $m^2\ g^{-1}$ for Cu/PI-300, PI-300, Cu/PI-400 and Cu/PI-600, respectively. The surface area of PI-300 support was not obviously changed after the deposition of Cu nanoparticles.

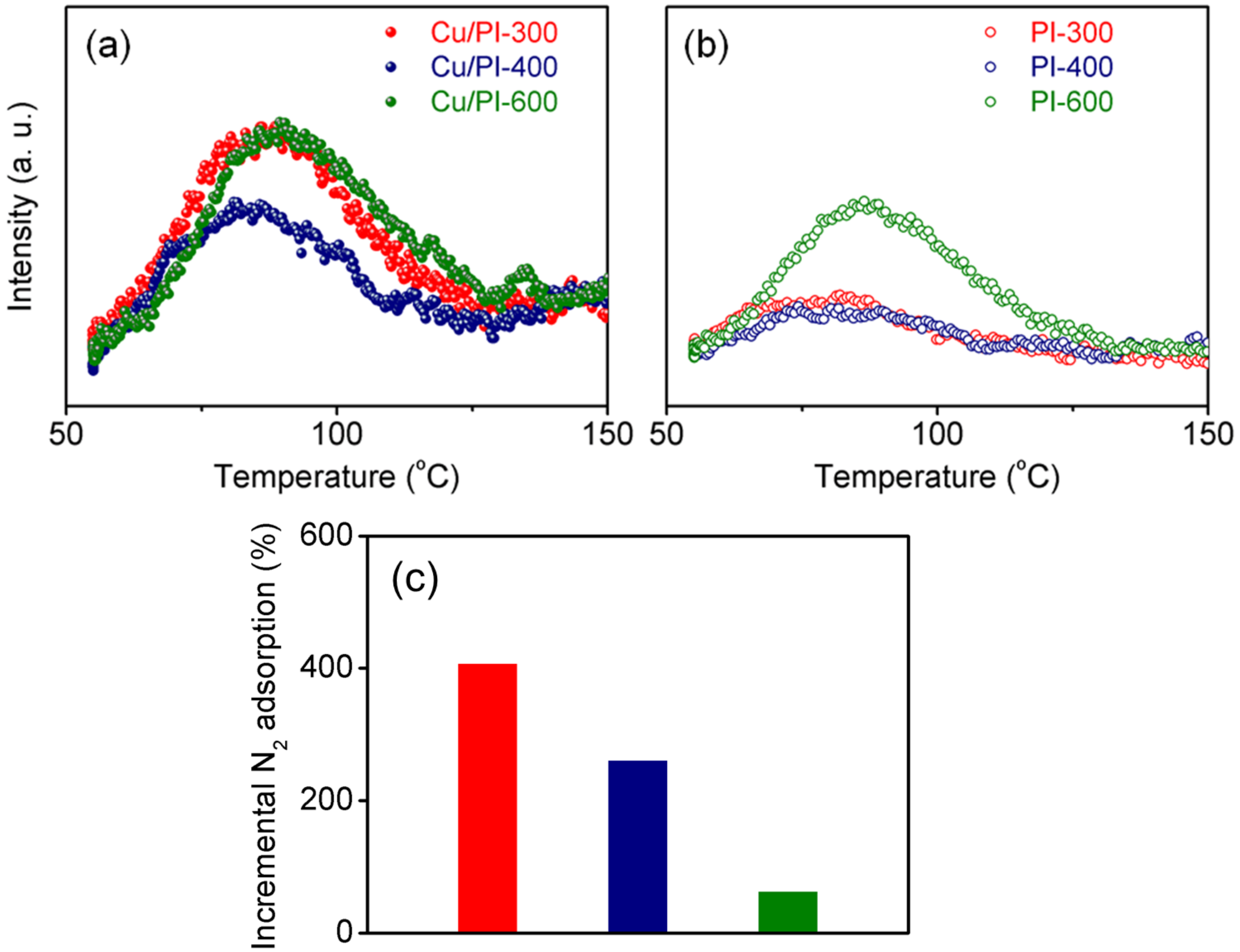

Supplementary Figure 25. (a) N_2-TPD curves of Cu/PI catalysts and (b) bare PI samples. The incremental N_2 adsorption column (c) showed that more pronounced electron-deficiency of Cu nanoparticles from Cu/PI-600 via Cu/PI-400 to Cu/PI-300 lead to gradually increased N_2 adsorption capacities for 0.6, 2.6 and 4.1 times as compared to the values of corresponding bare PI supports

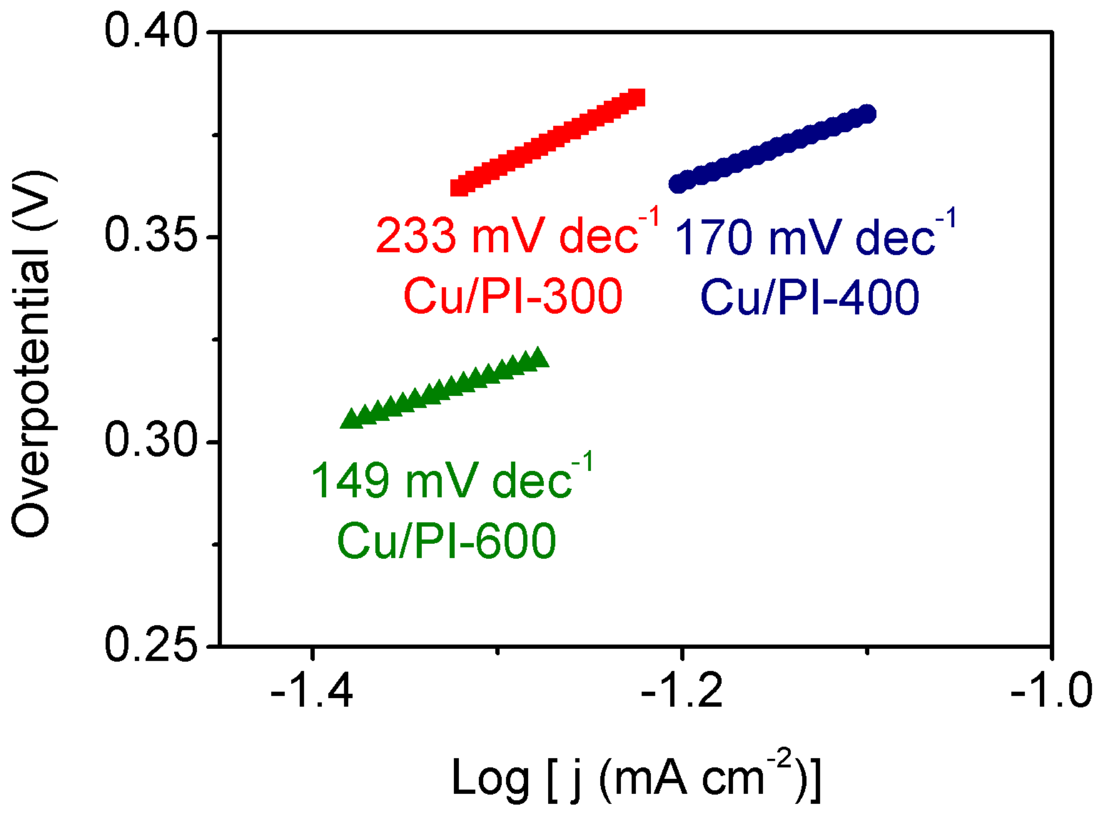

Supplementary Figure 26. The Tafel slopes of the Cu/PI catalysts calculated from corresponding LSV curves of HER measured in Ar-saturated electrolyte (1 M KOH). The Tafel slope value of Cu/PI-300 electrode (233 mV dec^{-1}) was higher than that of Cu/PI-400 electrode (170 mV dec^{-1}) and even higher than that of Cu/PI-600 electrode (149 mV dec^{-1}), indicating a less favorable HER kinetics process over the Cu/PI-300 electrode. Obviously, the electron-deficient effect on Cu will couple the OH^- on the interface of catalyst in basic electrolyte, inhibiting the HER route. Namely, the NRR process will be improved accordingly.

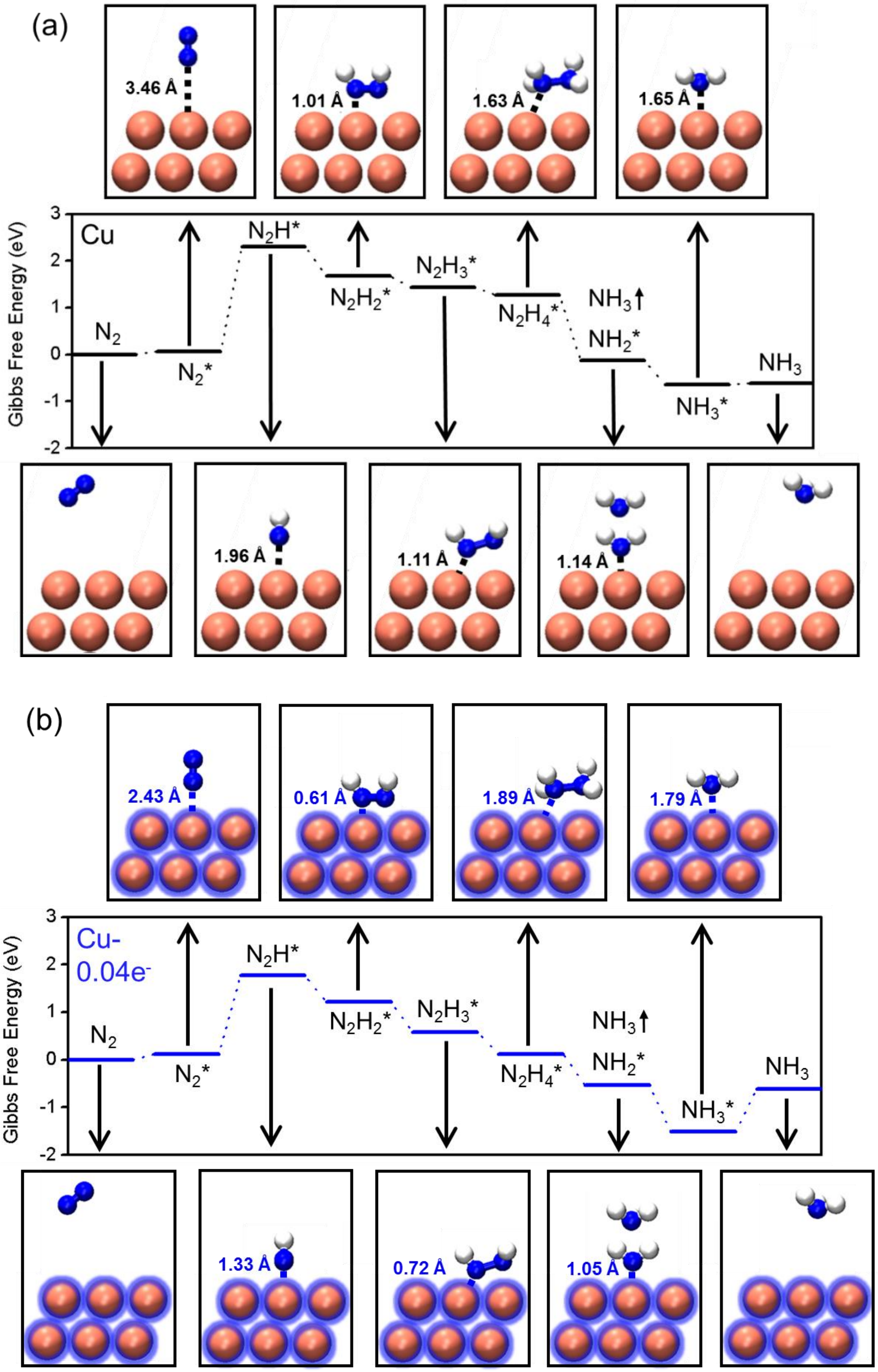
(a)
3.46 Å
1.01 Å
1.63 Å
1.65 Å
Cu
Gibbs Free Energy (eV)
N2
N2*
N2H*
N2H2*
N2H3*
N2H4*
NH3↑
NH2*
NH3*
NH3
1.96 Å
1.11 Å
1.14 Å
(b)
2.43 Å
0.61 Å
1.89 Å
1.79 Å
Cu-
0.04e-
1.33 Å
0.72 Å
1.05 Å

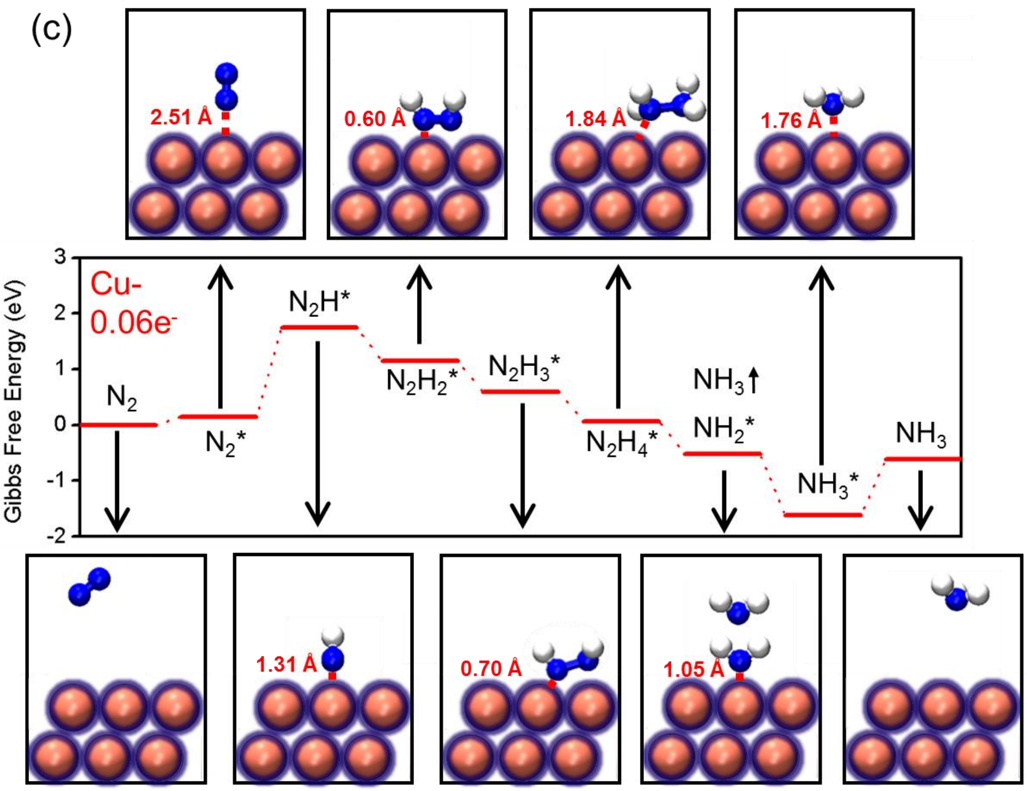

Supplementary Figure 27. Calculated absorption configurations and corresponding Gibbs free energy diagrams of each step of NRR process on (a) pristine Cu, (b) Cu-0.04e$^-$ and (c) Cu-0.06e$^-$ model.

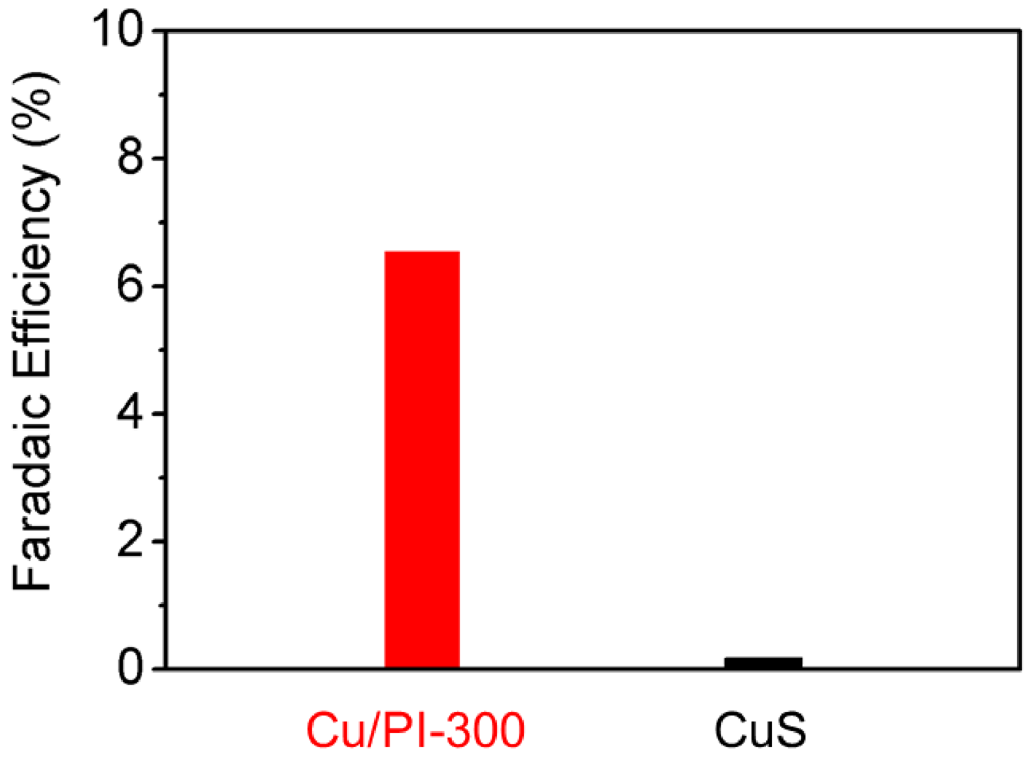

Supplementary Figure 28. The Faradaic efficiency of Cu/PI-300 and reported CuS.[S10]

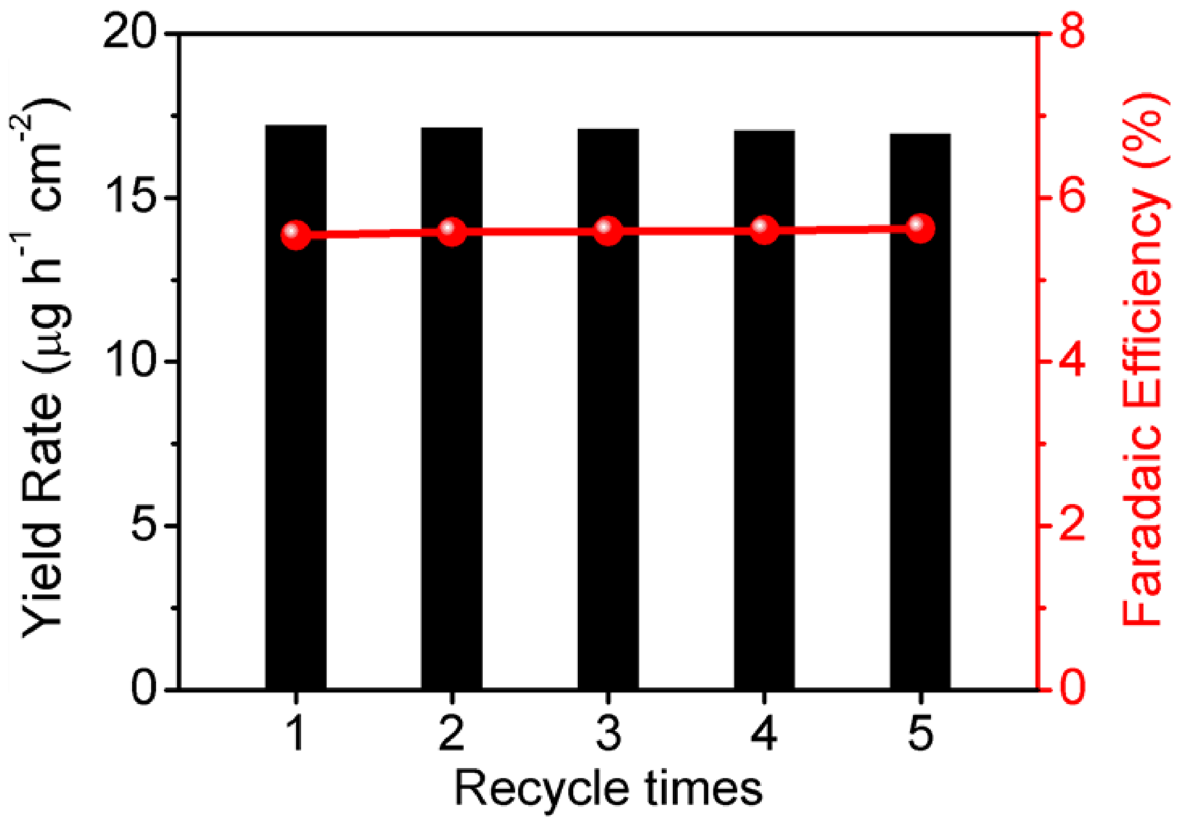

Supplementary Figure 29. The NH_3 yield rates and Faradaic efficiencies of the used Cu/PI-300 electrode during 5 cycles of 6-h NRR tests at -0.4 V vs RHE. The used electrolyte was replaced into fresh electrolyte before each reaction.

Supplementary Table 1. The Cu content of Cu/PI catalysts determined via the inductively coupled plasma (ICP) emission method.

**Sample**	**Cu/PI-300**	**Cu/PI-400**	**Cu/PI-600**	**Cu/C**
**Cu (wt.%)**	5.116±0.12	4.921±0.15	4.827±0.09	4.996±0.05

Supplementary Table 2. The NRR Faradaic efficiencies and NH_3 yield rates of Cu/PI-300, bare PI-300, Cu/C, CuO_x/PI-300 with a fixed catalyst loading (5 mg cm^{-2}) on carbon cloth electrodes at -0.3 V vs. RHE within 6 h.

**Sample**	**Cu/PI-300**	**PI-300**	**Cu/C**	**CuO_x/PI-300**	**Carbon cloth**
**Faradaic efficienciy (%)**	6.56	0	0.17	0.04	0
**NH_3 yield rate (μg h^{-1} cm^{-2})**	12.4	0	0.7	0.09	0

Carbon cloth (current collector) and bare PI-300 based electrodes were totally inert for NRR under standard conditions. Cu/C and CuO_x/PI-300 based electrodes were mainly inert for NRR under standard conditions.

Supplementary Table 3. Electrocatalytic NRR performance of the best-in-class Cu/PI-300 electrode and reported electrodes in aqueous electrolyte at ambient conditions.

Catalysts	Active loading (mg cm^{-2})	Electrolyte	Faraday efficiency (%)	NH_3 generation rate (μg h^{-1} cm^{-2})	Turnover frequency (h^{-1})	Ref.
**Cu/PI-300**	0.25	0.1 M KOH	6.56	17.2	0.26	This study
**CuS**	15	1.0 M KOH	0.18	7.62	--	S10
**Fe_2O_3**	1.4	Diluted $KHCO_3$	0.15	28.8	0.19	S11
**Ni wire**	6 cm^2	0.1 M LiCl/EDA	17.2	12.2	0.13	S12
**Au nanorod**	0.27	1 M KOH	4	1.65	0.07	S1
**Ru**	4.6	2 M KOH	0.28	0.208	0.0006	S13
**C-ZIF**	0.81	0.1M KOH	10.2	57.8	0.05	S14
**NPC**	0.6	0.05 M H_2SO_4	1.42	14.28	0.0168	S15
**Au/TiO_2**	1	0.1 M HCl	8.11	21.4	0.247	S16
**PEBCD/Li^+**	1.28	0.5 M Li_2SO_4	2.91	2	0.02	S17

Supplementary References:

S1. D. Bao *et al. Adv. Mater.* **29**, 1604799 (2017).

S2. J. P. Perdew, K. Burke, M. Ernzerhof, *Phys. Rev. Lett.* **77**, 3865-3868 (1996).

S3. B. Delley, *J. Chem. Phys.* **92**, 508-517 (1990).

S4. J. H. Montoya *et al. ChemSusChem* **8**, 2180-2186 (2015).

S5. Q. Sun *et al. J. Am. Chem. Soc.* **135**, 8246-8253 (2013).

S6. Z. Song, H. Zhan, Y. Zhou *Angew. Chem.* **122**, 8622-8626 (2010).

S7. B. Baumgartner, M. J. Bojdys, M. M. Unterlass *Polym. Chem.* **5**, 3771-3776 (2014).

S8. L. Han *et al. Angew. Chem. Int. Ed.* **58**, 2321 (2019).

S9. W. Feng *et al. Nanoscale* **11**, 1379-1385 (2019).

S10. N. Furuya, H. Yoshiba *J. Electroanal. Chem.* **291**, 269-272 (1990).

S11. S. Chen *et al. Angew. Chem., Int. Ed.* **56**, 2699-2703 (2017).

S12. K. Kim *et al. J. Electrochem. Soc.* **163**, 1523-1526 (2016).

S13. V. Kordali, G. Kyriacou, C. Lambrou, *Chem. Commun.* **17**, 1673-1674 (2000).

S14. S. Mukherjee *et al. Nano Energy* **48**, 217-226 (2018).

S15. Y. Liu *et al. ACS Catal.* **8**, 1186-1191 (2018).

S16. M. M. Shi *et al. Adv. Mater.* **29**, 1606550 (2017).

S17. G. F. Chen *et al. J. Am. Chem. Soc.* **139**, 9771-9774 (2017).
